# Changes in colon gene expression associated with increased colon inflammation in interleukin-10 gene-deficient mice inoculated with *Enterococcus *species

**DOI:** 10.1186/1471-2172-11-39

**Published:** 2010-07-15

**Authors:** Matthew PG Barnett, Warren C McNabb, Adrian L Cookson, Shuotun Zhu, Marcus Davy, Bianca Knoch, Katia Nones, Alison J Hodgkinson, Nicole C Roy

**Affiliations:** 1 Food, Metabolism & Microbiology Section, AgResearch Grasslands, Tennent Drive, Palmerston North 4442, New Zealand; 2 Food & Textiles Group, AgResearch Grasslands, Tennent Drive, Palmerston North 4442, New Zealand; 3 Riddet Institute, Massey University, Palmerston North, NZ; 4 Faculty of Medical and Health Sciences, The University of Auckland, 85 Park Road, Grafton, Auckland 1023, New Zealand; 5 Plant & Food Research, Ruakura Research Centre, East Street, Hamilton 3214, New Zealand; 6 Institute of Food, Nutrition & Human Health, Massey University, Tennent Drive, Palmerston North 4442, New Zealand; 7 Plant & Food Research, Batchelar Road, Palmerston North 4442, New Zealand; 8 Dairy Science & Technology, AgResearch Ruakura, East Street, Hamilton 3214, New Zealand

## Abstract

**Background:**

Inappropriate responses to normal intestinal bacteria may be involved in the development of Inflammatory Bowel Diseases (IBD, e.g. Crohn's Disease (CD), Ulcerative Colitis (UC)) and variations in the host genome may mediate this process. IL-10 gene-deficient (*Il10*^*-/-*^) mice develop CD-like colitis mainly in the colon, in part due to inappropriate responses to normal intestinal bacteria including *Enterococcus *strains, and have therefore been used as an animal model of CD. Comprehensive characterization of changes in cecum gene expression levels associated with inflammation in the *Il10*^*-/- *^mouse model has recently been reported. Our aim was to characterize changes in colonic gene expression levels in *Il10*^*-/- *^and C57BL/6J (C57; control) mice resulting from oral bacterial inoculation with 12 *Enterococcus faecalis *and *faecium *(EF) strains isolated from calves or poultry, complex intestinal flora (CIF) collected from healthy control mice, or a mixture of the two (EF·CIF). We investigated two hypotheses: (1) that oral inoculation of *Il10*^*-/- *^mice would result in greater and more consistent intestinal inflammation than that observed in *Il10*^*-/- *^mice not receiving this inoculation, and (2) that this inflammation would be associated with changes in colon gene expression levels similar to those previously observed in human studies, and these mice would therefore be an appropriate model for human CD.

**Results:**

At 12 weeks of age, total RNA extracted from intact colon was hybridized to Agilent 44 k mouse arrays. Differentially expressed genes were identified using linear models for microarray analysis (Bioconductor), and these genes were clustered using GeneSpring GX and Ingenuity Pathways Analysis software. Intestinal inflammation was increased in *Il10*^*-/- *^mice as a result of inoculation, with the strongest effect being in the EF and EF·CIF groups. Genes differentially expressed in *Il10*^*-/- *^mice as a result of EF or EF·CIF inoculation were associated with the following pathways: inflammatory disease (111 genes differentially expressed), immune response (209 genes), antigen presentation (11 genes, particularly major histocompatability complex Class II), fatty acid metabolism (30 genes) and detoxification (31 genes).

**Conclusions:**

Our results suggest that colonic inflammation in *Il10*^*-/- *^mice inoculated with solutions containing *Enterococcus *strains is associated with gene expression changes similar to those of human IBD, specifically CD, and that with the EF·CIF inoculum in particular this is an appropriate model to investigate food-gene interactions relevant to human CD.

## Background

The term 'Inflammatory Bowel Disease' (IBD) refers to a heterogeneous collection of conditions characterized by chronic inflammation of the gastrointestinal tract, and includes Crohn's Disease (CD) and Ulcerative Colitis (UC) [1]. While there is some overlap in disease pathology, CD and UC also have distinct pathologic features; CD can, for example, affect any part of the gastrointestinal tract, whereas UC is confined to the colon and rectum, often causing diarrhea. The inflammation seen in CD is typically discontinuous, segmental and involves all layers of the intestinal wall. In UC, inflammation tends to be continuous and superficial, only affecting the mucosal layer of the colonic wall [2].

The exact etiology and pathogenesis of IBD is still unclear, although there is strong epidemiological evidence for a genetic contribution to disease susceptibility. Several candidate genes for IBD susceptibility have been identified, including nucleotide-binding oligomerization domain containing 2 (NOD2) [3-5], tumour necrosis factor (TNF) [6], members of the toll-like receptor (TLR) family [7], IL-4 [8] and IL-18 [9], and a number of genes encoding transporter molecules, such as the ATP-binding cassette, sub-family B (MDR/TAP), member 1 (ABCB1) [10,11] and solute carrier family 22 (organic cation/ergothioneine transporter), member 4 (SLC22A4) genes [12,13].

The IL-10 gene deficient (*Il10*^*-/-*^) mouse has been used as a model of IBD [14-21]. These mice, when bred onto a C57BL/6J (C57) background, have been reported to develop CD-like colitis by 12 weeks of age when raised under conventional conditions [19], while female 129 Ola × C57*Il10*^*-/- *^mice have been shown to develop colitis from 20 weeks of age under specific pathogen free (SPF) conditions [21].

The precise mechanism that results in inflammation in *Il10*^*-/- *^mice is unclear, although, as is the case in human IBD, there is evidence of an inappropriate inflammatory response to normal intestinal flora [22].

Clinical isolates of *Enterococcus faecalis *have been shown to induce IBD-like symtoms in germ-free *Il10*^*-/- *^mice [14,23,24]. *Enterococcus *species are a common component of the intestinal flora of healthy humans and animals [25-27], comprising up to 1% of the adult microflora [28]. *Enterococcus faecalis *and *Enterococcus faecium *are the two species most commonly detected in the human bowel [29-31], and both are known to carry a variety of virulence factors (reviewed in [25]) which may play a role in the establishment of inflammation.

Based on these published studies, and on our own observations of only mild inflammation in 12 week old *Il10*^*-/- *^mice (C57 background) that were raised under conventional conditions (M. P. G. Barnett, "unpublished observations"), we decided to establish bacterially-inoculated *Il10*^*-/- *^mice as a model of IBD in order to test food-gene interactions associated with IBD. We tested two hypotheses: (1) that oral inoculation of *Il10*^*-/- *^mice with a mixture of pure *Enterococcus *isolates (both *faecalis *and *faecium*), alone or combined with conventional intestinal flora derived from healthy and conventionally raised C57 mice, would result in greater and more consistent intestinal inflammation than that observed in *Il10*^*-/- *^mice not receiving this inoculation, and (2) that this inflammation would be associated with changes in colon gene expression levels in key pathways similar to those previously observed in human studies, and these mice would therefore be an appropriate model for human CD [32,33].

We have previously published body weight, histology and preliminary gene expression data using this mouse model [34]. Here we describe in detail gene expression changes in colonic tissue in response to bacterial inoculation in *Il10*^*-/- *^on a C57 background, measured using high density oligonucleotide microarrays.

## Results

### Animal body weight

There was no difference in mean body weight between *Il10*^*-/- *^and C57 mice at the start of the experiment (*Il10*^*-/- *^18.9 ± 1.0 g; C57 18.8 ± 0.8 g). As reported previously [34], *Il10*^*-/- *^mice in the conventional conditions (C) and EF·CIF groups gained less weight during the course of the trial, both when compared to similarly-inoculated C57 mice, and when compared to *Il10*^*-/- *^mice in the other three treatment groups.

### Intestinal Histology

**Figure 1:**
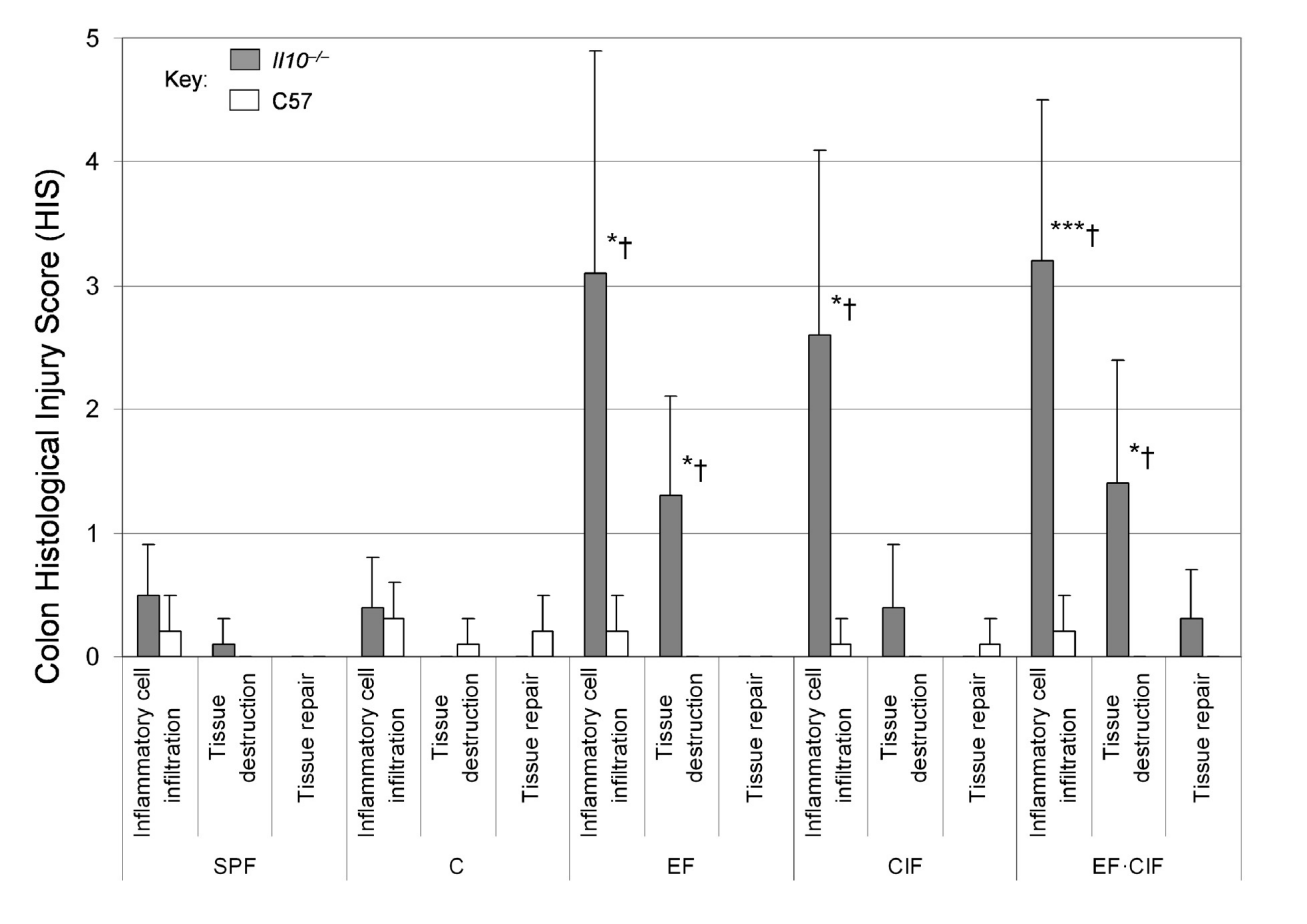
**Summary of the colon histological injury score (HIS) in *Il10*^*-/- *^and C57 mice in response to various treatments**. Data represent mean ± SD for 5 mice fed the AIN-76A diet, and housed in specific pathogen free (SPF) or conventional (C) conditions, or housed in conventional conditions and orally inoculated with 12 Enterococcus strains (EF), with conventional intestinal flora from normal C57 mice (CIF), or a 1:1 combination (EF·CIF). Asterisks denote *Il10*^*-/- *^mice with values significantly different (*P < 0.05, ***P < 0.01) from C57 mice in the same treatment group, while the "†" symbol denotes *Il10*^*-/- *^mice with values significantly (P < 0.05) different from *Il10*^*-/- *^mice under SPF conditions for the same histological parameter. One *Il10*^*-/- *^mouse in the CIF treatment group died during the course of the trial. An autopsy was unable to unequivocally establish the cause of death.

Total intestinal histology results have been reported elsewhere, in which colon was shown to be the intestinal section most susceptible to inflammation [34]. A more detailed analysis of the colon histology results showed that inflammatory cell infiltration was the most prominent feature of the observed inflammation in inoculated *Il10*^*-/- *^mice (accounting for approximately 80% of total colon HIS), and was significantly higher in these animals, when compared with C57 mice in the same group, or when compared with non-inoculated *Il10*^*-/- *^mice (i.e., SPF and C groups). Furthermore, *Il10*^*-/- *^mice inoculated with EF inocula (either EF or EF·CIF) showed significantly higher tissue destruction, both when compared with similarly-inoculated C57 mice, and when compared with *Il10*^*-/- *^under SPF and C conditions (Figure 1).

### Plasma Serum Amyloid A (SAA)

Overall, plasma SAA levels were higher in *Il10*^*-/- *^mice compared with C57 mice (*Il10*^*-/- *^87 ± 100; C57 15 ± 42 μg/ml, P < 0.001), and this difference between mouse strains was also significant in each of the treatment groups (P < 0.05). The interaction between strain and group was not significant (P = 0.53), and no overall significant within-strain effect of treatment group was detected (P = 0.15). While the lack of a significant between-strain difference in mice inoculated with EF·CIF seems to be due to the presence of two outliers (one in the C57 group and one in *Il10*^*-/- *^group), there is no data from any other observations which would justify the removal of these values. These data are summarized in Table 1.

**Table 1:** Plasma serum amyloid A and cytokine data from Il10^-/- ^and C57 mice

		Mouse strain		***P *(*Il10***^***-/-***^***vs*. C57)**
Assay	Treatment Group	***Il10***^***-/-***^	C57	
SAA	SPF	60.1 ± 30.4	7.1 ± 6.6	< 0.05
	C	43.9 ± 24.4	6.5 ± 4.7	< 0.05
	EF	140.5 ± 136.3	6.4 ± 7.5	< 0.05
	CIF	43.5 ± 11.4	6.5 ± 14.2	< 0.05
	EF·CIF	137.4 ± 161.1	50.6 ± 90.9	< 0.05
				
IL-1α	SPF	40.4 ± 39.6	25.0 ± 34.6	NS
	C	11.3 ± 25.2	38.3 ± 36.3	NS
	EF	^a ^80.3 ± 67.5	9.5 ± 21.2	0.03
	CIF	0.0 ± 0.0	55.2 ± 59.4	NS
	EF·CIF	44.9 ± 49.1	55.4 ± 59.6	NS
				
IL-4	SPF	1.0 ± 1.0	1.8 ± 1.1	NS
	C	1.0 ± 0.9	1.0 ± 0.5	NS
	EF	^b ^0.3 ± 0.6	^b ^0.5 ± 0.4	NS
	CIF	1.0 ± 1.9	1.0 ± 0.5	NS
	EF·CIF	^c ^0.4 ± 0.5	^c ^0.8 ± 1.6	NS
				
IL-5	SPF	33.4 ± 0.6	59.6 ± 57.0	NS
	C	19.9 ± 18.2	33.2 ± 1.7	NS
	EF	31.1 ± 19.3	35.9 ± 3.3	NS
	CIF	55.4 ± 68.6	26.7 ± 15.0	NS
	EF·CIF	22.1 ± 20.4	^d ^13.2 ± 18.1	NS
				
IL-6	SPF	2.4 ± 3.3	0.0 ± 0	NS
	C	5.2 ± 11.5	6.1 ± 6.8	NS
	EF	^e ^59.7 ± 30.0	5.4 ± 8.4	0.03
	CIF	8.0 ± 16.0	5.9 ± 8.9	NS
	EF·CIF	25.3 ± 24.5	0.03 ± 0.08	0.03

Data represent mean ± SD of plasma levels of SAA and IL-6 for 5 mice (except for *Il10*^*-/- *^mice in the CIF group, n = 4) fed AIN-76A diet, and in treatment groups described in the legend to Table 3. P-values are for across-group comparisons between *Il10*^*-/- *^and C57 mice under the same treatment conditions (NS denotes not significantly different between strains). Superscript letters denote mice with values significantly different (P < 0.05) from mice of the same strain in different treatment groups as follows: (a) EF-inoculated *Il10*^*-/- *^mice showed significantly higher IL-1α levels than *Il10*^*-/- *^mice in the C and CIF groups; (b) IL-4 in the EF groups was significantly lower than in the SPF and C groups in both mouse strains; (c) IL-4 in the EF·CIF group was significantly lower than that in the SPF group in both mouse strains; (d) across the C57 mice, those receiving the EF·CIF inoculation had significantly lower IL-5 levels than either EF or SPF; (e) *Il10*^*-/- *^mice in the EF inoculation group had significantly higher IL-6 than all other *Il10*^*-/- *^treatment groups except for EF·CIF.

A small number of SAA values were negative (i.e. were at the limit of detection). Because these data were log transformed (which resulted in homogeneous variance), a constant was added to all values before transformation. This constant was 6.8, being the absolute value of the minimum recorded value (-6.3 μg/ml) plus 0.5.

### Plasma Cytokines

For five of the cytokines measured (namely IL-10, IFNγ, TNFα, granulocyte monocyte colony-stimulating factor (GM-CSF), and IL-17), there were four or fewer non-zero values across the dataset, thus no statistical analysis was performed. Data from analysis of the remaining five plasma cytokines are summarized in Table 1.

### Microarrays

**Figure 2:**
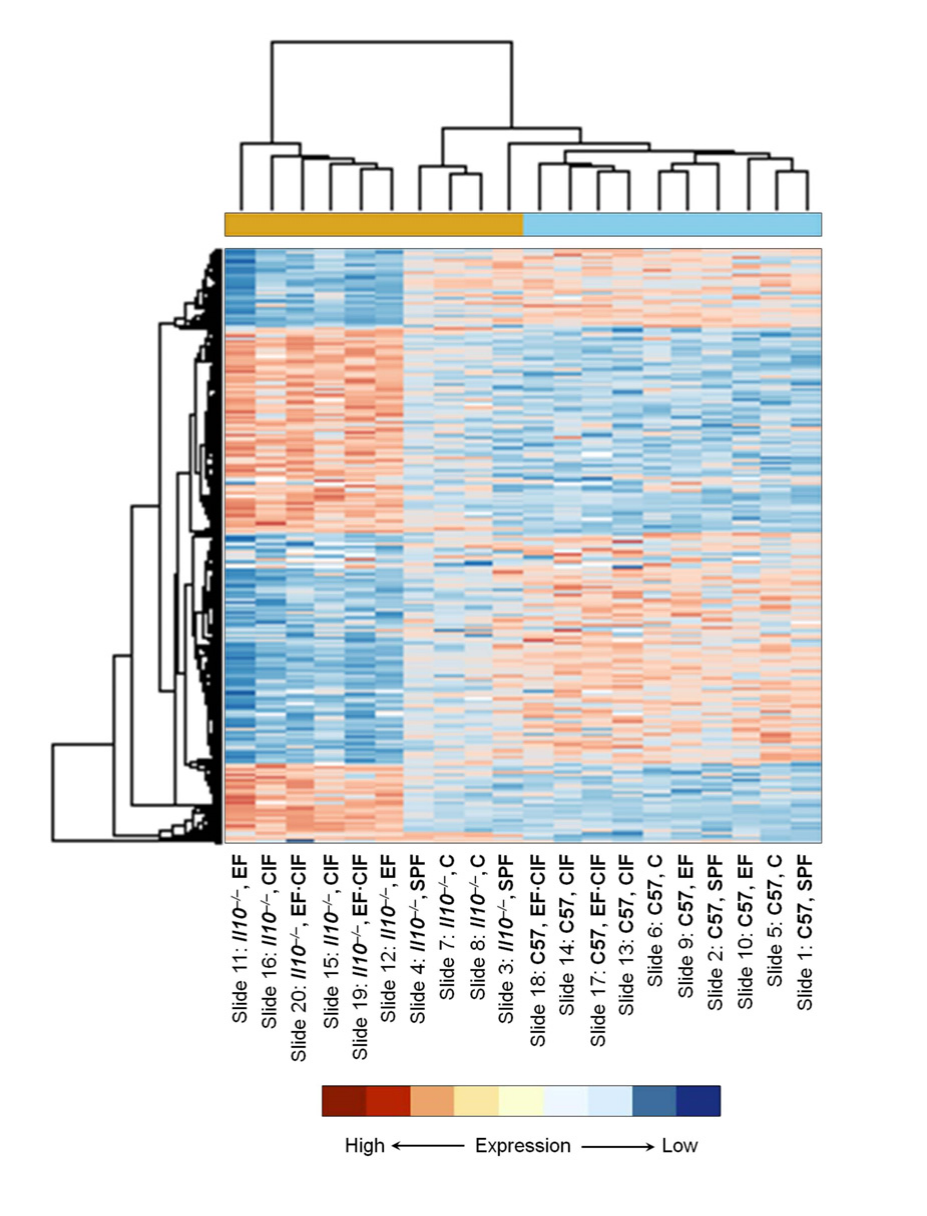
**Hierarchical clustering of 20 microarray slides (representing RNA pooled from either two or three mice per slide) and 6,521 probe sets**. A heat map of 6,521 transcripts and 2 dendograms that group gene probes (left) and microarray slides (top) together is shown. This represents all differentially expressed probes for the comparison *Il10*^-/- ^*vs*. C57 mice for any of the five treatment groups (SPF, C, EF, CIF, EF·CIF). Each line is a probe, and each column is an array slide. Expression signal intensities are shown in red and blue, indicating high and low expression, respectively. Arrays for *Il10*^-/- ^mice are represented in yellow and C57 mice in blue in the bar above the heat map.

We have previously described microarray data from these experiments comparing *Il10*^*-/- *^mice with C57 mice in each of the five treatment groups [34]. The total number of probes differentially expressed in any of the within-treatment (*Il10*^*-/- *^*vs*. C57) comparisons was 6,521, and this set was used for unsupervised hierarchical clustering. This analysis grouped the array slides into two main clusters: inoculated *Il10*^*-/- *^in one cluster, and C57 mice and non-inoculated *Il10*^*-/- *^mice in the other cluster (Figure 2).

We therefore decided to focus on genes differentially expressed when comparing inoculated *Il10*^*-/- *^mice (i.e. CIF, EF, and EF·CIF) with *Il10*^*-/- *^mice not receiving an inoculation, in order to better understand interactions or relationships between genes which may be leading to the increased inflammation observed in the inoculated mice (first hypothesis), and to compare these changes with those previously observed in human IBD (second hypothesis).

There were no significant differences in expression (*q *< 0.05) of any probes between *Il10*^*-/- *^mice in the C or SPF groups at 12 weeks of age, thus subsequent analysis of gene expression changes in inoculated *Il10*^*-/- *^mice was with those in the C group. *Il10*^*-/- *^in the CIF group had approximately 1,500 gene probes differentially expressed compared to those in group C, while *Il10*^*-/- *^mice inoculated with EF or EF·CIF had over 3,500 differentially expressed probes when compared with *Il10*^*-/- *^mice in group C (Table 2).

**Table 2:** Number of differentially expressed genes in colon tissue of Il10^-/- ^mice compared to C57 mice

	Differentially expressed genes (*q *< 0.05)			Ingenuity Pathways Analysis		
	
Comparison	Increase	Decrease	Total	Network eligible	Fold-change cut-off	Functions/pathways/lists eligible
*Between strain comparisons(Il10*^*-/- *^*vs. C57)*						
SPF	76	46	122	45	N/A	38
C	115	70	185	90	N/A	80
EF	2,258	2,565	4,823	2,494	|1.70|	740
CIF	1,630	1,845	3,475	1,136	|1.42|	736
EF·CIF	2,111	2,074	4,185	2,138	|1.47|	740
						
*Within strain comparisons (Il10*^*-/-*^*)*						
EF *vs*. C	1,602	2,028	3,630	1,085	|1.36|	746
CIF *vs*. C	756	734	1,490	463	N/A	421
EF·CIF *vs*. C	1,572	2,231	3,808	1,030	|1.36|	723

Data represent numbers of differentially expressed genes for each comparison, calculated using BioConductor 'limma analysis' package. False discovery rate control of *q *< 0.05 was used as a filtering criterion, and the fold-change cut-off listed applied to each group to reduce the Network Eligible genes for Ingenuity Pathways Analysis to 800 ± 1. The most significant functions were identified using Ingenuity Pathway Analysis Software. Total RNA from colon tissue was used for microarray hybridization to Agilent Technologies 44 k (G4122-60510) mouse 60 mer oligonucleotide arrays. Data represent mean values from two pools of RNA for each mouse strain, with RNA from either two or three mice in each pool.

### Comparison with human IBD

Thirty two genes are known to undergo changes in transcriptional activity in IBD [35]. In order to assess the relevance of the inoculation protocols used in our study to that of human disease, we compared changes in expression of these thirty-two genes across each of the treatment groups. These results, summarised in Table 3, show that the two inoculations containing EF strains were most effective in mimicking human IBD on the basis of gene expression changes, in particular with respect to changes in gene expression levels of cytokines and chemokines and their receptors.

**Table 3:** Differentially expressed genes in colon tissue of *Il10*^*-/- *^mice compared to C57 mice, and in *Il10*^*-/- *^mice orally inoculated with intestinal bacteria, in common with those observed in human studies

Gene	RefSeq ID	Mouse Model								
		
		Transfer	***Between strain comparisons (Il10***^***-/- ***^***vs. C57)***					***Within strain comparisons (Il10***^***-/-***^***)***		
			SPF	C	EF	CIF	EF·CIF	EF vs C	CIF vs C	EF·CIF vs C
*Cytokine and cytokineR genes:*										
Tnf	NM_013693	*2.3*	1.1	1.1	*2.4 *^*c*^	*2.0 *^*c*^	*2.3 *^*c*^	*1.9 *^*c*^	*1.7 *^*b*^	*2.0 *^*c*^
Ifng	NM_008337	*4.7*	1.2	1.2	*3.2 *^*c*^	*2.1 *^*c*^	*2.4 *^*c*^	*2.3 *^*c*^	*1.9 *^*b*^	*2.0 *^*c*^
Ltb	NM_008518	*8.9*	2.0	1.4	*3.1 *^*c*^	*2.5 *^*c*^	*2.8 *^*c*^	*2.1 *^*b*^	*2.0 *^*b*^	*2.3 *^*b*^
Il6	NM_031168	*2.6*	-1.0	1.0	1.2	1.1	1.2	1.3	1.3	1.4
Il16	NM_010551	*2.7*	1.1	1.1	*1.4 *^*a*^	1. 1	1.2	1.3	1.2	1.2
Il18R1	NM_008365	*17.7*	1.2	1.2	*1.5 *^*b*^	*1.6 *^*b*^	1.2	*1.3 *^*a*^	1.3	1.2
Il22	NM_016971	*9.3*	ND	ND	ND	ND	ND	ND	ND	ND
*Chemokine and chemokineR genes:*										
Ccr2	NM_009915	*7.1*	*1.6 *^*a*^	*1.6 *^*a*^	*2.6 *^*c*^	*2.7 *^*c*^	*1.9 *^*c*^	*1.8 *^*c*^	*1.8 *^*b*^	*1.7 *^*b*^
Ccr7	NM_007719	*2.2*	1.0	1.0	-1.2	1.3	1.2	-1.1	1.3	1.1
Ccl2	NM_011333	*2.5*	1.6	1.4	*4.5 *^*c*^	*3.6 *^*c*^	*3.6 *^*c*^	*3.1 *^*c*^	*2.5 *^*c*^	*2.8 *^*c*^
Ccl3	NM_011337	*4.1*	-1.0	1.2	1.6	1.6	*2.4 *^*a*^	1.3	1.2	*1.9 *^*a*^
Ccl4	NM_013652	*4.3*	1.2	1.1	*1.9 *^*b*^	*1.7 *^*a*^	*2.2 *^*b*^	*1.8 *^*a*^	1.7	*2.0 *^*b*^
Ccl5	NM_013653	*6.1*	1.7	1.0	*5.1 *^*c*^	*1.7 *^*a*^	*2.1 *^*b*^	*3.0 *^*c*^	*3.0 *^*c*^	*3.5 *^*c*^
Ccl7	NM_013654	*3.1*	1.2	1.1	*1.8 *^*b*^	*1.5 *^*a*^	*1.6 *^*c*^	*1.7 *^*b*^	1.5	*1.7 *^*b*^
Ccl11	NM_011330	1.4	1.1	-1.1	-1.0	1.0	1.1	1.1	1.1	*1.3 *^*a*^
Ccl17	NM_011332	*2.4*	1.1	1.1	*1.3 *^*a*^	*1.4 *^*a*^	1.1	1.2	1.1	1.0
Ccl20	NM_016960	*10.4*	1.1	1.0	1.2	*1.4 *^*a*^	1.2	1.1	1.3	1.2
Cxcr3	NM_009910	*2.0*	1.2	-1.0	*1.6 *^*b*^	1.2	*1.4 *^*a*^	*1.5 *^*a*^	1.3	*1.5 *^*a*^
Cxcl1	NM_008176	*4.8*	1.0	1.1	*2.4 *^*c*^	*1.7 *^*b*^	*2.3 *^*c*^	*1.8 *^*b*^	1.4	*1.9 *^*b*^
Cxcl5	NM_009141	*21.9*	1.4	1.3	*3.4 *^*c*^	*2.5 *^*c*^	*2.8 *^*c*^	*2.2 *^*c*^	*2.1 *^*b*^	*2.4 *^*c*^
Cxcl10	NM_021274	*14.7*	1.6	1.6	*6.9 *^*c*^	*6.0 *^*c*^	*7.2 *^*c*^	*3.4 *^*b*^	*2.7 *^*a*^	*2.8 *^*a*^
*Genes involved in tissue remodeling:*										
Mmp3	NM_010809	*28*	1.3	1.1	*3.7 *^*c*^	*3.1 *^*c*^	*4.0 *^*c*^	*2.9 *^*c*^	*2.6 *^*b*^	*3.8 *^*c*^
Mmp7	NM_010810	*5.3*	1.4	1.3	*2.3 *^*c*^	*2.0 *^*c*^	*2.2 *^*c*^	*1.6 *^*b*^	1.4	*1.6 *^*b*^
Mmp9	NM_013599	*2.0*	1.0	1.1	1.3	1.2	1.2	1.1	1.1	1.1
Mmp14	NM_008608	*2.7*	1.1	1.2	*1.5 *^*a*^	1.3	1.2	1.1	1.2	1.3
Timp1	NM_011593	1.4	1.2	1.0	1.2	1.3	1.3	1.2	1.3	1.3
*Regenerating islet derived genes:*										
Reg3g	NM_011260	*221.8*	*5.3 *^*a*^	*9.0 *^*b*^	*3.4 *^*a*^	*8.8 *^*c*^	*5.7 *^*b*^	-1.7	-1.8	-2.2
Pap (Reg3b)	NM_011036	*132.1*	*8.0 *^*b*^	*4.4 *^*a*^	1.9	*5.2 *^*b*^	2.4	-1.7	-1.8	-2.2
*S-100 family genes:*										
S-100a8	NM_013650	*133.1*	1.4	1.2	*6.5 *^*c*^	*5.8 *^*c*^	*9.0 *^*c*^	*4.7 *^*c*^	*4.2 *^*c*^	*7.1 *^*c*^
S-100a9	NM_009114	*225.4*	1.6	1.7	*13.8 *^*c*^	*15.7 *^*c*^	*22.8 *^*c*^	*9.2 *^*c*^	*9.5 *^*c*^	*14.2 *^*c*^
*Multidrug resistance (MDR) genes:*										
Abcb1a	NM_011076	*-8.2*	-1.3	*-2.5 *^*a*^	*-5.7 *^*c*^	*-3.6 *^*c*^	*-4.5 *^*c*^	*-2.3 *^*b*^	-1.6	*-2.1 *^*b*^
*Genes involved in epithelial metabolism and biosynthesis:*										
Ptgs2	NM_011198	*3.2*	1.1	1.1	1.3	1.1	1.4	1.3	1.1	1.4
										
***Total DE genes:***		**30**	**4**	**4**	**22**	**21**	**19**	**11**	**18**	**19**

Data represent differentially expressed genes for each comparison in common with the 32 genes shown to be differentially expressed in human IBD, with the "Transfer" column showing data from a CD45RB Transfer Colitis model previously published by te Velde *et al *[35]. Total RNA from colon tissue was used for microarray hybridization to Agilent Technologies 44 k (G4122-60510) mouse 60 mer oligonucleotide arrays. Data represent mean values from two pools of RNA for each mouse strain, with RNA from either two or three mice in each pool. In the case of significantly differentially expressed genes, the text is shown in italics, and denoted by superscript letters where p < 0.05 is represented by ^a^, p < 0.01 by ^b^, and p < 0.001 by ^c^. The total number of differentially expressed genes in each comparison (maximum 32) is shown at the bottom of the table.

### Ingenuity pathways analysis

Table 4 shows the top functions in each treatment group and the number of differentially expressed genes associated with each function. For all groups analyzed using IPA, the most significant biological functions were grouped into three categories: 1) Diseases and Disorders; 2) Molecular and Cellular Functions; and 3) Physiological System Development and Function (Table 4).

**Table 4:** Key functions associated with inflammation in Il10-/- mice identified using Ingenuity Pathways Analysis

Top Function	Number of differentially expressed genes (*P*-value)				
	SPF	C	EF	CIF	EF·CIF
*Diseases and Disorders*					
Immunological Disease	9 (< 0.05)	9 (< 0.05)	176 (< 0.0001)	127 (< 0.0005)	117 (< 0.0005)
Inflammatory Disease	7 (< 0.05)	9 (< 0.05)	180 (< 0.0001)	112 (< 0.0005)	113 (< 0.0005)
Cancer	6 (< 0.05)	9 (< 0.05)	339 (< 0.0001)	249 (< 0.0005)	242 (< 0.0005)
Organismal injury and abnormality	7 (< 0.05)	11 (< 0.05)	95 (< 0.0001)	61 (< 0.0005)	63 (< 0.0005)
Haematological Disease	3 (< 0.05)	4 (< 0.05)	139 (< 0.0001)	58 (< 0.0005)	107 (< 0.0005)
*Molecular and Cellular Function*					
Cell-to-cell Signaling and Interaction	14 (< 0.05)	11 (< 0.05)	188 (< 0.0001)	156 (< 0.0005)	155 (< 0.0005)
Cellular Movement	5 (< 0.05)	3 (< 0.05)	192 (< 0.0001)	156 (< 0.0005)	164 (< 0.0005)
Amino Acid Metabolism	2 (< 0.05)	2 (< 0.05)	N/A	72 (< 0.0005)	78 (< 0.0001)
Carbohydrate Metabolism	2 (< 0.05)	9 (< 0.05)	59 (< 0.0001)	102 (< 0.0005)	113 (< 0.0005)
Cell Death	7 (< 0.05)	10 (< 0.05)	250 (< 0.0001)	221 (< 0.0005)	221 (< 0.0005)
*Physiological System Development and Function*					
Immune Response	22 (< 0.05)	18 (< 0.05)	190 (< 0.0001)	190 (< 0.0005)	186 (< 0.0005)
Immune and lymphatic system development	16 (< 0.05)	16 (< 0.05)	169 (< 0.0001)	150 (< 0.0005)	150 (< 0.0005)
Hematological system development and function	15 (< 0.05)	14 (< 0.05)	190 (< 0.0001)	179 (< 0.0005)	171 (< 0.0005)
Tissue morphology	7 (< 0.05)	6 (< 0.05)	134 (< 0.0001)	108 (< 0.0005)	114 (< 0.0005)
Tissue development	9 (< 0.05)	1 (< 0.005)	122 (< 0.0001)	78 (< 0.0005)	80 (< 0.0005)

Table 5 lists the Canonical Pathways showing significant differences between *Il10*^*-/- *^and C57 mice within each treatment group, calculated by IPA. Calculation was either according to ratio (the number of genes from the data set that map to the canonical pathway in question divided by the total number of genes that map to the same canonical pathway) or significance (Fischer's exact test was used to calculate a P-value determining the probability that the association between the genes in the dataset and the canonical pathway is explained by chance alone). A full list of the genes within the ten most significant Canonical Pathways is shown in Additional file 1 Table S1.

**Table 5:** Key canonical pathways associated with inflammation in *Il10*^*-/- *^mice

Canonical Pathways	Number of differentially expressed molecules (*P*-value)				
	SPF	C	EF	CIF	EF·CIF
*Ranked according to ratio (differentially expressed genes/total number of genes)*					
Antigen Presentation Pathway	5/39 (< 0.0001)	5/39 (< 0.0001)	10/39 (< 0.0001)	10/39 (< 0.0001)	10/39 (< 0.0001)
Interferon Signaling	1/29 (0.1)	1/29 (0.1)	9/29 (< 0.0001)	6/29 (< 0.005)	6/29 (< 0.01)
IL-10 Signaling	1/70 (0.2)	2/70 (< 0.05)	12/70 (< 0.0001)	12/70 (< 0.001)	13/70 (< 0.0005)
p38 MAPK Signaling	1/95 (0.3)	2/95 (0.1)	13/95 (< 0.001)	15/95 (< 0.0005)	17/95 (< 0.0001)
Cell Cycle: G2/M DNA Damage Checkpoint Regulation	N/A	N/A	7/42 (< 0.01)	5/42 (0.06)	7/42 (< 0.01)
Inositol Metabolism	N/A	N/A	4/24 (< 0.005)	3/24 (0.13)	4/24 (< 0.05)
Keratan Sulfate Biosynthesis	N/A	N/A	5/31 (< 0.05)	3/31 (026)	5/31 (< 0.05)
IL-6 Signaling	N/A	1/93 (0.41)	13/93 (< 0.0005)	13/93 (< 0.005)	15/93 (< 0.0005)
Fatty Acid Metabolism	1/187 (0.5)	4/187 (< 0.05)	28/187 (< 0.0001)	26/187 (< 0.0001)	28/187 (< 0.0001)
*Ranked according to P-value (as determined by IPA)*					
Total number of significant pathways	2	6	15	44	40
Fatty Acid Metabolism	1/187 (0.3)	2/187 (0.1)	28/187 (< 0.0001)	25/187 (< 0.0001)	28/187 (< 0.0001)
LPS/IL-1 Mediated Inhibition of RXR Function	1/170 (0.4)	2/170 (0.2)	33/170 (< 0.0001)	26/170 (< 0.0001)	31/170 (< 0.0001)
Tryptophan Metabolism	2/237 (0.05)	1/237 (0.4)	26/237 (< 0.0001)	22/237 (< 0.0001)	24/237 (< 0.0001)
β-alanine Metabolism	N/A	N/A	10/99 (< 0.0001)	15/99 (< 0.0001)	14/99 (< 0.0001)
Valine, Leucine and Isoleucine Degradation	N/A	N/A	12/107 (< 0.0001)	14/107 (< 0.0005)	16/107 (< 0.0001)

In order to examine those molecules directly interacting with IL-10 (and therefore gain insight into the effect of the lack of this protein in the *Il10*^*-/- *^mice), the Neighbourhood Explorer function of IPA was used. The Network Neighborhood of IL-10 consists of 405 molecules which have been identified as directly interacting with IL-10. The number of differentially expressed genes (*Il10*^*-/- *^*vs*. C57) within this network as a result of the treatments was as follows: SPF (7); C (8); EF (86); CIF (79); EF·CIF (82). Two genes of interest in this comparison were regenerating islet-derived 3 beta (*Reg3b*) (Table 3) and the polymeric immunoglobulin receptor (*pIgR*), as these were the only genes which showed significantly higher expression in *Il10*^*-/- *^mice in the SPF, C and CIF groups (compared to C57 mice), but for which there was no difference in the EF and EF·CIF groups. The expression of *Reg3b *mRNA was between 4- and 8-fold higher in the colon of *Il10*^*-/- *^mice in the SPF and C and CIF groups when compared to C57 mice in the same groups, but there was no differential expression of this molecule in the EF and EF·CIF groups (Table 3). In the case of the related *Reg3g *gene, while its expression was higher in *Il10*^*-/- *^mice in all experimental groups, the difference was greatest in the C and CIF groups (~9-fold), less in the SPF and EF·CIF groups (~5-fold) and lowest in the EF group (3-fold, Table 3). In the case of *pIgR*, expression was higher in the SPF (2.0-fold), C (2.2-fold) and CIF (1.7-fold) groups when comparing *Il10*^*-/- *^mice with C57 mice, but there was no difference in the EF or EF·CIF groups.

**Figure 3:**
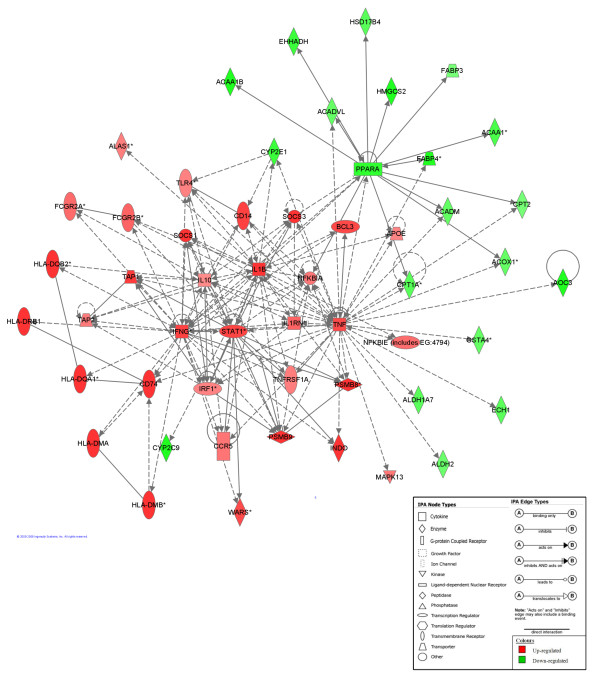
**Generation of a biological network of genes of the most significant Canonical Pathways for the EF·CIF (Il10-/- vs. C57) comparison**. The network was generated by IPA using all molecules from significantly affected Canonical Pathways (Fatty Acid Metabolism; LPS/IL-1 mediated inhibition of RXR function; Tryptophan Metabolism; β-Alanine Metabolism; Valine, Leucine and Isoleucine Degradation; Antigen Presentation Pathway; Interferon Signaling; IL-10 Signaling; Fatty Acid Elongation in Mitochondria). Connections were applied based on known interactions between these genes within the Ingenuity Pathways Knowledge Base. Central genes and their direct interactions were identified and were supported by published information. *Genes that are detected 2 or more times on the array. Genes or gene products are represented as nodes, and the biological relationship between two nodes is represented as a line (i.e. an edge). All edges are supported by at least 1 reference from the literature. Red and green colored nodes indicate genes with up- and down-regulated expression, respectively. The intensity of the colors specifies the degree of up- or down-regulation. Greater intensity represents a higher level of differential expression. Nodes and edges are displayed with various shapes and labels that present the functional class of genes and the nature of the relationships between the nodes, as shown in legend below the figure.

**Figure 4:**
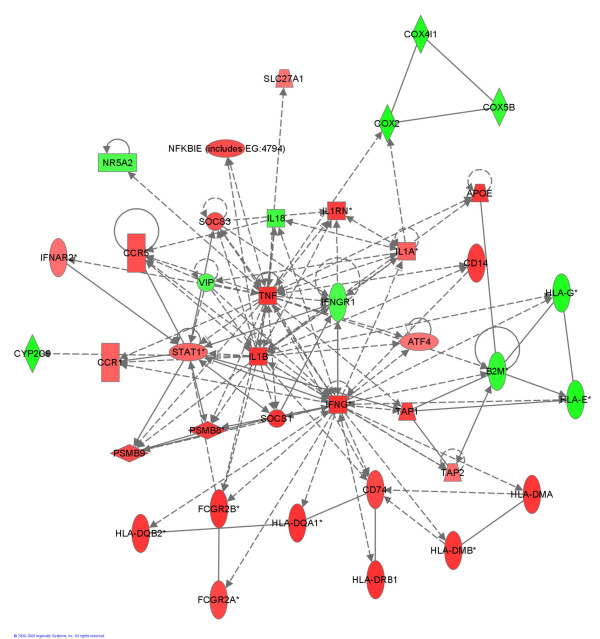
**Generation of a biological network of genes of the most significant Canonical Pathways for the Il10-/- (EF·CIF vs. C) comparison**. The network was generated by IPA using all molecules from significantly affected Canonical Pathways (Oxidative Phosphorylation; Antigen Presentation Pathway; IL-10 Signaling; Interferon Signaling; LPS/IL-1 mediated inhibition of RXR function; Circadian Rhythm Signaling). Connections were applied based on known interactions between these genes within the Ingenuity Pathways Knowledge Base. Central genes and their direct interactions were identified and were supported by published information. *Genes that are detected 2 or more times on the array. Genes or gene products are represented as nodes, and the biological relationship between two nodes is represented as a line (i.e. an edge). All edges are supported by at least 1 reference from the literature. Red and green coloured nodes indicate genes with up- and down-regulated expression, respectively. The intensity of the colours specifies the degree of up- or down-regulation. Greater intensity represents a higher level of differential expression. Nodes and edges are displayed with various shapes and labels that present the functional class of genes and the nature of the relationships between the nodes, as shown in the legend to Figure 3.

In order to better understand the effect of the EF·CIF inoculation in *Il10*^*-/- *^mice compared with the same inoculation in C57 mice, genes from the top five canonical pathways on the basis of IPA P-value (Oxidative Phosphorylation, Antigen Presentation Pathway, IL-10 Signaling, Interferon Signaling, LPS/IL-1 mediated inhibition of RXR function) were combined with those from the top five on the basis of calculated ratio (Antigen Presentation Pathway, Interferon Signaling, Oxidative Phosphorylation, IL-10 Signaling, Circadian Rhythm Signaling). A network was built by connecting these molecules according to their interactions, as determined by the IPA Knowledge base (Figure 3). A similar analysis was performed for EF·CIF-inoculated *Il10*^*-/- *^mice compared to *Il10*^*-/- *^mice which were not inoculated (*Il10*^*-/- *^(EF·CIF *vs*. C), Figure 4).

Key molecules in the *Il10*^*-/- *^(EF·CIF *vs*. C) comparison were: IFNγ, TNF, STAT1, IL-1β and suppressor of cytokine signaling 1 (SOCS1) and SOCS3. These molecules also appear to be important when comparing EF·CIF-inoculated *Il10*^*-/- *^with C57 receiving the same inoculation (Figure3), as do peroxisome proliferator-activated receptor alpha (PPARα) and TLR4. Conversely, the expression levels of the pro-inflammatory IL-18 gene were reduced in the *Il10*^*-/- *^(EF·CIF *vs*. C) comparison, and in Figure 4, IL-18 is shown to interact directly with five other molecules in the network; in contrast, this molecule does not feature in the EF·CIF (*Il10*^*-/- *^*vs*. C57) comparison (Figure 3).

### Quantitative real-time polymerase chain reaction (qRT-PCR)

**Figure 5:**
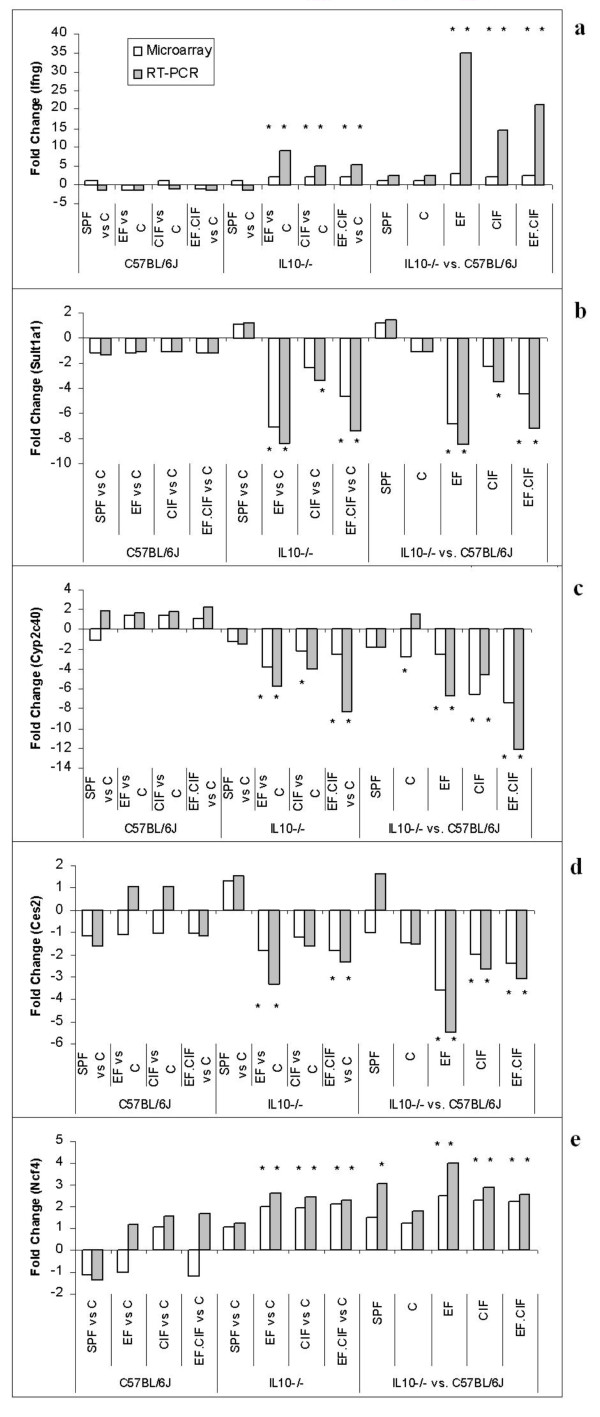
**Real time quantitative PCR validation of gene expression results from microarray analysis**. Real time quantitative PCR was performed to determine the relative expression of *Ifng *(a), *Sult1a1 *(b), *Cyp2c40 *(c), *Ces2 *(d) and *Ncf4 *(e), five genes which showed differential expression (as determined using microarray analysis) in *Il10*^*-/- *^mice as a result of bacterial inoculation. Results for the differentially expressed genes were normalized against the geometric mean of CANX and HPRT. An asterisk indicates a significant difference in gene expression for the comparison of interest.

For the three xenobiotic metabolism genes selected for qRT-PCR validation (*Cyp2c40*, *Ces2 *and *Sult1a1*) the reduced expression in inoculated *Il10*^*-/- *^mice observed in the microarray analysis was confirmed (both magnitude and direction) by qRT-PCR. In the case of the immune response genes *Ifng *and *Ncf4*, the direction of the gene expression change (an increase in both cases) was confirmed, but the magnitude of the change for *Ifng *was higher in the PCR analysis compared to the microarray results (Figure 5).

## Discussion

Our study clearly shows that oral inoculation of 5 week old *Il10*^*-/- *^mice (C57 background) with a bacterial preparation (12 *Enterococcus *strains combined with complex intestinal flora from C57 mice raised under conventional conditions) results in consistent and increased colon inflammation at 12 weeks of age, compared with *Il10*^*-/- *^mice which were not inoculated (C group). These tissue changes were similar to those seen in human IBD, and were accompanied by significant changes in expression of genes involved in 'immune response', 'inflammatory diseases' and 'antigen presentation'.

### Histological analysis

Several studies have reported the development of intestinal inflammation in *Il10*^*-/- *^mice when raised under either SPF or conventional conditions in the absence of bacterial inoculation [16,19,36]. It has been also reported that the induction of IBD-like colitis (and therefore relevance as a model) in the *Il10*^*-/- *^mouse is dependent on the background mouse strain (with C3H/HeJBir or BALB/C *Il10*^*-/- *^mouse being more susceptible, and C57BL/6 *Il10*^*-/- *^mice (the strain used in this study) being more resistant to the development of intestinal inflammation [1,19]) and the commensal bacteria to which the mice are exposed [23]. We observed no significant difference in intestinal inflammation between *Il10*^*-/- *^mice (C57 background) and C57 control mice when kept in either SPF or conventional conditions. In addition, comparison of colon gene expression levels between *Il10*^*-/- *^and C57 mice in these two treatment groups showed relatively few changes in the current study. The *Il10*^*-/- *^mice in these studies were brought in from The Jackson Laboratory (where they had been raised under SPF conditions), were free of all tested viruses, bacteria and mycoplasma and other opportunistic organisms, and were maintained under quarantine during the trial. It therefore seems likely that the failure of these mice to develop inflammation under conventional conditions is due at least in part to insufficient exposure to commensal intestinal bacteria. Our results clearly demonstrate that, in the absence of such bacterial exposure and in the conventional conditions used in our study, the *Il10*^*-/- *^mouse on a C57 background at 12 weeks of age is not a suitable model for human IBD.

### Relevance of bacterial inoculation

All of the inoculation protocols we tested resulted in increased intestinal (particularly colon) inflammation in *Il10*^*-/- *^mice, both when compared with similarly-inoculated C57 mice and when compared with *Il10*^*-/- *^mice which received no inoculation (SPF or C groups). Although the CIF inoculation showed some evidence of colon inflammation, based on HIS the two inoculation preparations containing *Enterococcus *species (EF and EF·CIF) were more effective in the induction of inflammation. In particular, the EF·CIF inoculation resulted in a significant increase in overall HIS, with the greatest effect observed in the colon. The nature of the inflammation within the colon was transmural and discontinuous, affecting different layers of the intestinal wall, similar to human CD [2]. Our findings for the *Il10*^*-/- *^mice inoculated with *Enterococcus *species are in agreement with those of other studies where these bacterial species have been shown to induce inflammation in *Il10*^*-/- *^mice [14,23,37]. Our results support observations that inoculation of *Il10*^*-/- *^mice with *Enterococcus *species gives rise to characteristics of chronic inflammatory diseases, such as a dysregulated immune response acting in combination with inflammatory mechanisms that may lead to tissue damage.

This study used *Enterococcus *strains isolated from calves and poultry, and there were several reasons for this. First, these strains were readily available, and there is clear evidence in the literature that *Enterococcus *species induce inflammation in this mouse model [14,23,24]; establishing a relevant mouse model of IBD within our laboratory was the key goal of these studies. Furthermore, enterococci are ubiquitous as a gastrointestinal bacterium in warm-blooded animals, and using those strains isolated from calves ensured that we included enterococci from a mammalian host. Lastly, all strains were fecal isolates, and therefore derived from an appropriate environment.

We do not have specific information on the relationship of these isolates to those derived from mice. It is possible that some virulence factors in poultry may be different from calves, which may be different from mice. However, we have shown that the enterococci, either alone or in combination with CIF derived from control mice, trigger inflammation regardless of the fact that they are from a different host. Thus the original host may be immaterial, and there is some factor (or factors) common to *Enterococcus *strains which triggers inflammation in a genetically predisposed host. Other studies have used human oral *Enterococcus *isolates in *Il10*^*-/- *^mice [23,37], which also suggests that there is no host specificity.

Finally, while the ability of the bacteria within the various inocula to colonize, and to persist, within the murine gut was not established in this study, neither colonisation nor persistence is necessarily a pre-requisite if the appropriate inflammatory response is observed even from transiently dosed enterococci. Thus, although the long-term fate of the inoculated bacteria within the murine gut was not established, the consequences of the inoculation in terms of intestinal inflammation, which was the key outcome of the study, have been clearly established.

### Gene expression profile in inoculated *Il10*^*-/- *^mice

Each of the inoculation procedures induced changes in the expression levels of a large number of genes, both when comparing *Il10*^*-/- *^mice with similarly inoculated C57 mice and when comparing the inoculated *Il10*^*-/- *^mice with those raised under conventional conditions.

It is apparent from the unsupervised hierarchical clustering analysis of all differentially expressed genes (Figure2) that the various groups of mice fall into two main clusters, with C57 mice and *Il10*^*-/- *^mice in the SPF and C groups (i.e. those which had not received any inoculation) in one, and inoculated *Il10*^*-/- *^mice in the other. The *Il10*^*-/- *^mice that did not receive any inoculation were clustered more closely with the C57 mice than with those *Il10*^*-/- *^mice which did receive a bacterial inoculation. This confirms the histological observation that, in the absence of bacterial inoculation, the *Il10*^*-/- *^mouse on a C57 background at 12 weeks of age is not a suitable model for human IBD in the conditions used for our study. Furthermore, bacterial inoculation led to many more changes in the expression of genes that would normally interact with IL-10.

Of the three inoculation protocols, CIF appears to be the least effective in triggering intestinal inflammation, showing the lowest number of relevant differentially expressed genes. This is in agreement with published studies showing *Enterococcus *species to be particularly effective in triggering inflammation in the *Il10*^*-/- *^mouse model.

Expression levels of mRNA encoding pro-inflammatory cytokines IFN-γ, TNF-α, and IL-1 in colon tissue were higher in inoculated *Il10*^*-/- *^mice compared with C57 mice, regardless of the inoculation, although mRNA expression of the pleiotropic pro-inflammatory cytokine IL-18 was lower in the colon of EF·CIF-inoculated mice. The pro-inflammatory IL-6 was detected in plasma samples at higher levels in both EF and EF·CIF-inoculated *Il10*^*-/- *^mice compared with similarly inoculated C57 mice and (in the case of EF) compared with non-inoculated *Il10*^*-/- *^mice, although there was no difference in the colon mRNA levels of IL-6 for these two inoculations as determined by microarray analysis. IL-6, a pleiotropic cytokine, has been shown to play a crucial role in the chronic inflammatory process in IBD [38]. This cytokine is produced by macrophages, lymphocytes and intestinal epithelial cells in response to intestinal microflora [39] and stimulates T-cell expansion via anti-apoptopic signaling [38]. T-cell accumulation increases IL-6 levels and thereby a vicious cycle is induced, leading to chronic inflammation. While there were also some differences in the concentrations of other plasma cytokines both within and across strains (e.g. IL-1α higher in EF-inoculated *Il10*^*-/- *^mice, IL-4 lower in EF- and EF·CIF-inoculated mice, both *Il10*^*-/- *^and C57), the levels of Th1/Th2 cytokines measured in plasma samples were generally low or undetectable. In future studies, inflammatory markers such as these Th1/Th2 cytokines could be measured at the site of inflammation to confirm observed gene expression changes, and further verify the relevance of this model of IBD. Our findings are in agreement with observations that the pathogenesis of IBD in humans is mediated in part through an imbalance of pro- and anti-inflammatory cytokines [40]. Our inoculated *Il10*^*-/- *^mice showed a cytokine profile (increased IFNγ, TNFα, IL-1, IL-6 gene expression levels in colon tissues) that is characteristic of the CD inflammatory response. CD is thought to be dominated by a T-helper (Th)-1 response [41], with increased production of IFNγ [42] and IL-2 [43]. TNF-α has been identified as a key pathogenic cytokine for immune-mediated inflammatory diseases such as CD [44].

As shown in Table 3, this is further supported by the fact that inoculation of *Il10*^*-/- *^mice with *Enterococcus *strains led to differential expression (compared with *Il10*^*-/- *^mice that did not receive an inoculation) of many of the thirty two genes (19 for EF·CIF, 18 for EF) previously identified in the literature as being relevant to IBD in human studies [35], including the ATP-binding cassette (ABC) transporter *ABCB1A*. Our study is also in agreement with other studies using *Il10*^*-/- *^mice, in which MHC class II mRNA expression levels in cecal samples was increased, and cytochrome-P450 expression was decreased [45]. We observed up-regulation of MHC class II molecule mRNA (antigen presentation pathway, Table 5) and down-regulation of mRNA expression of several cytochrome P450 genes (*Cyp4b1*, *Cyp2c9, Cyp2c18*), as well as a sulfotransferase (*Sult1c1*), a carboxylesterase (*Ces1*) and the ATP-binding cassette (ABC) transporters *Abcb1b, Abcb4, Abcb6 *and *Abcb9 *in the colon of EF·CIF-inoculated *Il10*^*-/- *^mice. Other xenobiotic metabolism genes such as aldo-keto reductases (*Akr1c6*), a flavin containing monooxygenase (*Fmo5*), and three of the genes selected for RT-PCR validation (*Ces2, Sult1a1 *and *Cyp2c40*) were also down-regulated. A decrease in the expression of detoxification enzymes and transporters may be an effect secondary to the occurrence of inflammation; both inflammation and infection are known to down-regulate the activity and expression levels of drug metabolizing enzymes and transporters [46].

Our results clearly show that expression of mRNA encoding the CD14 protein, a receptor expressed on the surface of macrophages, monocytes and neutrophils which is essential for LPS-dependent signal transduction via TLR4 [47], was up-regulated in *Il10*^*-/- *^mice as a result of bacterial inoculation, particularly associated with EF inoculation (both EF and EF·CIF treatments). This could be a mechanism by which inflammation is initiated in our mouse model of IBD. Quantitative trait loci and microarray analyses of intestinal tissues of two *Il10*^*-/- *^strains with divergent susceptibility to developing inflammation, C3H/HeJBir *Il10*^*-/- *^mice (most susceptible) and C57 *Il10*^*-/- *^mice (less susceptible), identified *Cd14 *as a candidate gene associated with this difference in susceptibility [1]. In addition, a polymorphism in the human CD14 promoter (resulting in increased expression of the CD14 receptor) was shown to be associated with both CD [48] and UC [49]. Both of these studies refer to genetic susceptibility to inflammation associated with CD14, while our study shows a change in the expression of *Cd14 *mRNA in inflamed mice, possibly caused by increased cell influx, thus they are not directly comparable. All of these studies do, however, highlight the potential importance of CD14 in the initiation of intestinal inflammation.

In the current study, *Reg3b *mRNA levels were higher in the colon of *Il10*^*-/- *^mice in the SPF and C (non-inflamed) and CIF (moderately inflamed) groups when compared to C57 mice in the same groups, but not in the EF and EF·CIF groups, which showed more severe signs of inflammation (Table 3); a similar pattern was observed for the *Reg3g *gene. *Reg3b *and *Reg3g *are expressed in mouse intestine [50], and encode murine orthologues of human pancreatitis-associated proteins (PAP) which may be involved in the innate immune response to bacterial colonisation of the intestinal tract [51] and can inhibit the inflammatory response by blocking NFκB activation [7].

The *Reg3b *and *Reg3g *genes are induced after development of intestinal inflammation in germ-free severe combined immunodeficiency (SCID) mice colonised with commensal bacteria [52], and bacterial-epithelial contact may drive *Reg3g *expression as a mechanism to limit microbial penetration and maintain mucosal surface integrity [53]. In our study, the fact that expression levels of both *Reg3b *and *Reg3g *were lower in *Il10*^*-/- *^mice inoculated with *Enterococcus *strains relative those in the C and CIF groups may reflect an inability of these mice to appropriately respond to this inoculation, and a failure to effectively deal with the additional enterococci introduced via inoculation [54]. Alternatively, lower overall expression levels of these two *Reg3 *genes in response to *Enterococcus *inoculation may result in insufficient suppression of the immune response, potentially a crucial early step in the initiation and development of intestinal inflammation in these mice. This is in agreement with studies in the *Il2*^*-/- *^mouse model, where increased expression levels of *Reg3b *and *Reg3g *genes might be associated with prevention of colitis triggered by colonization with commensal *E. coli *[51]. While the levels of *Reg3b *may be sufficient to explain the lack of phenotype in the SPF/C groups (and to a lesser extent the CIF group), the absence of IL10 (which has been shown to regulate *Reg3b *in rat cells in vitro [55]) in the *Il10*^*-/- *^mice may mean that insufficient *Reg3b *is expressed to suppress inflammation induced by enterococci. It is not clear from the literature what constitutes a 'suppressive' amount of *Reg3b *in a colitic phenotype, and further studies would be required to establish this, and to clarify functional roles of either *Reg3b *or *Reg3g *in *Enterococcus*-induced inflammation in this model.

The decreased expression of polymeric immunoglobulin receptor (*Pigr*) mRNA in *Il10*^*-/- *^mice in the EF and EF·CIF groups showed a similar pattern of expression to *Reg3b *in our study. The protein product of the *Pigr *gene is a glycoprotein expressed on the basolateral surface of secretory columnar and crypt epithelial cells, and is responsible for active transport of secretory antibodies such as IgA across the secretory epithelium that lines the mucosal surfaces. Studies with *Pigr *knockout (*Pigr*^*-/-*^) mice have produced strong evidence that innate secretory antibodies protect against invasion by pathogenic bacteria via immune exclusion; these mice are unable to bind and actively transport dimeric IgA to the mucosa and thus are more susceptible to infection with virulent strains such as *Salmonella *[56]. Furthermore, *Pigr*^*-/- *^mice exhibit profound immunopathological changes and clinical disease in response to induction of colitis with dextran sulfate sodium, suggesting that *Pigr *may play an important role in modulating inflammatory responses in the mucosa during active colitis [57]. The low *Pigr *gene expression in our *Il10*^*-/- *^mice receiving an inoculation with *Enterococcus *strains suggests a reduced capacity to prevent induction of a systemic immune response, a possible mechanism by which otherwise harmless commensal bacterial strains may have initiated the chronic inflammation observed in these two treatment groups.

In the current study, antigen presentation genes such as major histocompatibility complex (MHC) class II molecules (human leukocyte antigen (HLA) family members - *H2-Ab1*, *H2-Aa*, *H2-Eb1 *and *Cd74*) showed higher expression levels in colon in *Il10*^*-/- *^mice in all treatment groups (SPF, C, EF, CIF, EF·CIF) compared with C57 mice in the same treatment groups. The up-regulation of antigen presentation genes in our study is consistent with increased expression of HLA class II molecules that typically occurs in IBD in humans [58]. In addition, MHC class II antigen expression has been observed in the colon epithelial cells of *Il10*^*-/- *^mice as early as three weeks of age, with higher expression levels observed in mice at three to six months of age [19]. It has also been reported that IFNγ appears to play a role in development of intestinal colitis, but that neither IFNγ nor MHC class II expression are required for sustaining disease once it has become established [19]. In our study, in contrast to antigen presentation gene response, there was no difference in expression levels of colon *Tnf *or *Ifng *genes between *Il10*^*-/- *^and C57 mice in the C or SPF groups, but both of these genes were up-regulated in *Il10*^*-/- *^mice as a result of bacterial inoculation. This occurs when compared with similarly-inoculated C57 mice, and with *Il10*^*-/- *^mice in the C group. These results are further evidence that IFNγ does play a role in the development of intestinal colitis, whereas MHC class II expression alone is not sufficient to trigger an inflammatory response in this model.

## Conclusions

Overall, our findings indicate that inoculation of *Il10*^*-/- *^mice with solutions containing intestinal bacteria increases colon inflammation, and that the use of *Enterococcus *strains in particular results in a more appropriate model of IBD compared with non-inoculated *Il10*^*-/- *^mice. High density oligonucleotide microarrays have identified gene expression changes in *Il10*^*-/- *^colonic tissue in response to bacterial inoculation that are consistent with the current knowledge of mechanisms responsible for human IBD. In addition, there is preliminary evidence for the inflammatory response in this model being initiated by a failure of the normal mechanisms which recognize commensal bacterial, through molecules such as pIgR and REG3A/REG3G. Our data suggest that in particular the EF·CIF inoculation, which results in exposure to a highly complex bacterial environment, gives an appropriate and relevant mouse model of human IBD in which a variety of food components could be tested to establish potential ameliorating effects, and to understand the mechanisms by which these effects may occur.

## Methods

### Animals and diet

This study was reviewed and approved by the AgResearch Ruakura Animal Ethics Committee in Hamilton, New Zealand according to the Animal Protection Act (1960) and Animal Protection Regulations (1987) and amendments. Twenty five male *Il10*^*-/- *^(C57 background, formal designation B6.129P2-*Il10*^*tm1Cgn*^/J) mice and twenty five male C57 control mice were received from The Jackson Laboratory (Bar Harbor, ME, USA) at approximately 5 weeks of age. Prior to this time, *Il10*^*-/- *^mice were raised under SPF conditions, and were therefore free of all tested viruses, bacteria and mycoplasma and other opportunistic organisms (including *Helicobactor*, *Pasteurella*, and *Pseudomonas*). Mice were maintained under quarantine throughout the trial, and were housed either in pairs or groups of three (5 mice per treatment) in shoebox-style cages containing untreated wood shavings and a plastic tube for environmental enrichment. The animal room was maintained at a temperature of ~22°C and humidity of ~50% with a 12-hour light/dark cycle. All mice had *ad libitum *access to water, which was refreshed twice a week. An AIN-76A powdered diet, prepared as previously described [59], was supplied twice a week, with sufficient provided to meet the daily intake of *Il10*^*-/- *^mice, as determined in a previous feeding trial (data not shown). The diet for all groups was sterilized by gamma irradiation (25 kGy, Schering-Plough, Wellington, New Zealand) to a level required for SPF conditions, to minimize the possibility of bacterial introduction to the SPF group of animals. All mice were weighed twice weekly and carefully monitored for disease symptoms (weight loss, soft faeces and inactivity).

### Experimental design

**Figure 6:**
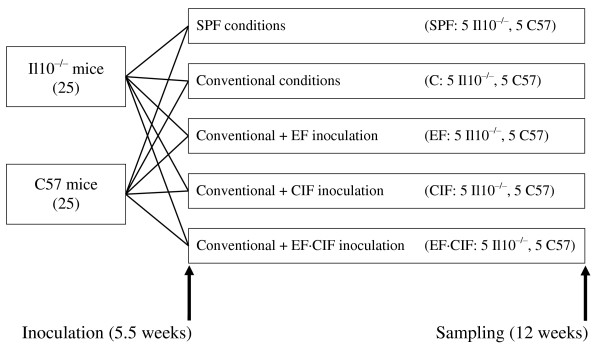
**Overall study design**. Both *Il10*^*-/- *^and C57 mice were fed AIN-76A diet from the time of arrival (34 ± 3 days of age), and were randomly divided into five treatment groups with five animals per group. One group of mice was housed in SPF conditions, a second group was maintained under conventional conditions (C), while the remaining groups were kept in conventional conditions and orally inoculated (200 μl) with solutions containing either the 12 strains of Enterococcus listed in Table 1 (EF), conventional intestinal flora (CIF) derived from healthy age-matched C57 mice raised under conventional conditions, or a combination of the two (EF·CIF). Inoculation was performed at approximately 5.5 weeks of age, and tissue sampling at 12 weeks of age.

Both *Il10*^*-/- *^and C57 mice were randomly divided into five treatment groups with five animals per group (Figure 6). One group of mice was housed in SPF conditions (isolator cages supplied with high efficiency particulate air (HEPA)- filtered air (Tecniplast SpA, Buguggiate, Italy)); a second group was maintained under conventional conditions (C), while the remaining groups were kept in conventional conditions and orally inoculated (200 μl) with solutions containing either the 12 strains of *Enterococcus *listed in Table 1 (EF; 1.2 × 10^8 ^colony forming units (CFU)), CIF derived from healthy age-matched C57BL/6 mice raised under conventional conditions, or a combination of the two (EF·CIF; 6.0 × 10^7 ^CFU from the EF inoculum), as described below. The CIF inoculation was included to better mimic the complete microbiota associated with the mouse gastrointestinal tract. Microbial ecology of the gastrointestinal tract is a complex interaction between microorganisms, the host and food components with potentially as few as 50% of microbes being able to be cultured in the laboratory, many requiring unknown or undefined conditions for growth [60]. It is likely that many of the uncultivable micro-organisms may have a role in gastrointestinal tract homeostasis in addition to eliciting inflammation.

### Preparation of bacterial solutions for oral inoculation

Enterococcus strains were obtained from calf or poultry faeces using sterile Amies transfer swabs (Biolab Limited, Auckland, New Zealand) and inoculating directly onto Slanetz and Bartley agar (Oxoid, Hampshire, UK). Plates were incubated at 42°C for 48 hours with red or purple colonies subcultured and incubated using the same culture conditions. Putative enterococcus strains were stored as glycerol stocks at -85°C in brain heart infusion broth supplemented with 30% (v/v) glycerol. Speciation of the *E. faecium *and *E. faecalis *strains was performed using PCR primers specific for amplification of the D-ala D-ala ligase gene - ddlEF (5'-TAGAGACATTGAATATGCC-3') and ddlER (5'-AATCGCACCGGCTCAATC-3') - which were modified from a previous study [61].

Seventy-two hours prior to inoculation, colonies of the *E. faecalis *and *E. faecium *strains (Table 6) were sub-cultured from slope tubes onto fresh Slanetz & Bartley agar, and incubated at 42°C for 48 h. A single colony from each culture was subsequently transferred to 5 ml of Todd Hewitt Broth (Oxoid, Hampshire, UK), and incubated at 37°C for 24 h. Each *Enterococcus *culture was centrifuged to pellet the bacterial cells (3000 *g*, 10 min, 4°C), which were then re-suspended in 5 ml sterile PBS (pH 7.4) and pooled (EF inoculum).

**Table 6:** Strains of *Enterococcus faecalis *and *E. faecium *used in solutions for oral inoculation

*Enterococcus faecalis*		*Enterococcus faecium*	
**Strain**	**Source**	**Strain**	**Source**

AGR991	calf	SN081	calf
AGR1140	calf	SN068	calf
AGR1371	calf	SN077	calf
SN070	poultry	SN067	calf
SN079	poultry	SN071	calf
SN083	poultry	AGR979	poultry

All strains were individually subcultured on Slanetz & Bartley medium, grown in Todd Hewitt Broth and re-suspended in 5 ml sterile PBS (pH 7.4) before pooling. Inoculation solutions used in each of the three groups of inoculated mice (Figure 6) were prepared as described in the text.

The healthy, age-matched C57BL/6 mice from which flora were being collected were euthanized by CO_2 _asphyxiation and cervical dislocation and the gastrointestinal tract (from stomach to just below the caecum) removed. Digesta were collected from the intestine and caecum by gently washing with sterile PBS, pH 7.4, then suspended in a total of 30 ml PBS. After mixing by gentle inversion and a settling period of approximately 5 min, the suspension was collected (CIF inoculum). Inoculation solutions EF and CIF were mixed in a 1:1 (v/v) solution to obtain the EF·CIF inoculum.

### Sample collection

At 12 weeks of age, samples were collected from all mice. To minimize time variation between the last food intake and sampling, mice were fasted overnight on the night before sampling. On the morning of sampling, food was returned for two hours, followed by a further two hour fast immediately prior to tissue sampling [62].

Mice from all treatments were euthanized using CO_2 _asphyxiation followed by cervical dislocation. Blood was sampled via cardiac puncture (0.5 to 1 ml), cells pelleted and the plasma snap-frozen and stored at -85°C for subsequent cytokine and SAA analysis. The intestine was quickly removed, cut open lengthwise and flushed with 0.9% sodium chloride to remove any traces of digesta, then carefully laid out on an ice-cold stainless steel tray. Sections of each intestinal region (duodenum, jejunum, ileum and colon) were frozen in liquid nitrogen before storage at -85°C, for gene profiling, and a sub-sample from each intestinal region was fixed in 10% phosphate buffered formalin and stored at room temperature until histological evaluation of inflammation.

### Histology

Histological examination was performed as previously described [34,59]. Briefly, formalin-fixed samples were processed, sectioned, stained with haematoxylin and eosin and evaluated for inflammation under a light microscope, using a modification of a previously described scoring system [15,63]. A histological injury score (HIS) was assigned based on the presence of inflammatory lesions, tissue destruction and tissue repair.

### Plasma SAA

Inflammation was also determined by analysis of SAA levels in plasma using a murine-specific Phase SAA ELISA kit, according to the manufacturer's protocol (Tridelta Development Ltd., Maynooth, County Kildare, Ireland). Pipetting of standards and samples was performed using an epMotion 5070 Liquid Handling Workstation (Eppendorf South Pacific Pty. Ltd., NSW, Australia). Briefly, standards or samples plus biotinylated monoclonal SAA antibody were incubated in microtitre plate wells pre-coated with capture monoclonal SAA antibody. In one step, SAA (in the standard or sample) was captured and labelled in a sandwich format. After washing to remove unbound material, wells were incubated with streptavidin-horseradish peroxidase prior to the addition of enzyme substrate (3,3',5,5'-tetramethylbenzidine, TMB). The reaction was stopped with the addition of 2 M sulphuric acid. Optical density in the wells was measured at 450 nm (630 nm as reference) using an automated plate reader (Versa_max_, Molecular Devices, CA, USA).

### Plasma cytokines

Plasma samples were analyzed for the presence of Th1/Th2-related cytokines (IL-1α, IL-2, IL-5, IL-6, IL-10, IFNγ, TNFα, granulocyte monocyte colony-stimulating factor (GM-CSF), IL-4 and IL-17) using a FlowCytomix Multiplex (Th1/Th2 10plex) mouse kit (Bender MedSystems GmbH, Vienna, Austria) according to the manufacturer's instructions. Briefly, microbeads coated with antibodies to the specified cytokines were mixed with plasma samples. Biotinylated secondary antibodies were then added, and the mixture incubated at room temperature for 2 h with constant shaking (500 rpm). The amount of cytokine that bound to the antibodies was then detected using streptavidin conjugated to phycoerythrin, with end-point measurement in a flow cytometer (FACScan, Becton Dickinson, North Ryde, NSW, Australia). Levels of each cytokine were determined by comparison with a standard curve (concentration range for all cytokines 0-20000 pg/ml).

### RNA isolation and synthesis of labeled cRNA

Total RNA from intact colon tissue was isolated by homogenizing the samples in TRIzol (Invitrogen, Auckland, New Zealand) according to the manufacturer's instructions. RNA was quantified with a Nanodrop ND-1000 spectrophotometer (NanoDrop Technologies, Wilmington, DE, USA), and RNA integrity was assessed with an RNA 6000 Nano LabChip kit using the Agilent 2100 Bioanalyser (Agilent Technologies, Palo Alto, CA, USA). Extracted RNA was purified using RNeasy spin columns (QIAGEN, San Diego, CA, USA). Only total RNA with an OD 260/280 ratio > 2.0, a Bioanalyzer 28 s/18 s peak ratio ≥ 1.2 and an RNA integrity number ≥ 7.5 was used for microarray hybridization. An equimolar pool of total RNA extracts from colon tissues of two or three different mice per treatment was made, resulting in two pools per treatment. A reference design was used for microarray hybridization: colonic RNA extracts from all mice were pooled in an equimolar proportion and used as the reference sample.

The Low RNA Input Fluorescent Linear Amplification Kit (Agilent Technologies Inc., Palo Alto, CA, USA) was used to synthesize cDNA and fluorescent cRNA. Labelled cRNA was made on the same day for all pools, including the reference sample. cDNA was synthesized from 500 ng of purified total RNA from each pool according to the manufacturer's protocol. Cyanine 3-cytidine triphosphate (Cy3, PerkinElmer, Waltham, Massachusetts 02451, USA) was used to label sample groups, while the reference RNA was labeled with cyanine 5-CTP (Cy5, PerkinElmer, Waltham, MA 02451, USA). Hybridization was performed according to a reference design without dye swap.

### Microarray hybridization and scanning

The *in situ *hybridization kit-plus (Agilent Technologies Inc., Palo Alto, CA, USA) was used to hybridize cRNA samples to Agilent Technologies Mouse G4121A - 44 k 60 mer oligo arrays. For each experimental group, two pools of cRNA were hybridized, thus in total twenty arrays were used. Cy3-labelled cRNA (0.75 μg) and Cy5-labelled cRNA (0.75 μg) were hybridised onto the microarray according to the manufacturer's protocol, as previously described [34]. Slides were scanned using a GenePix 4200A scanner (Molecular Devices Corporation, Sunnyvale, CA, USA) at a photomultiplier tube (PMT) setting of 450 V. Spot identification and quantification were performed using GenePix 6.0 software (Molecular Devices Corporation, Sunnyvale, CA, USA). The microarray data are available as accession GSE12223 in the Gene Expression Omnibus repository at the National Center for Biotechnology Information http://www.ncbi.nlm.nih.gov/geo/info/linking.html.

### Microarray data analysis

Statistical analysis was performed using linear models for microarray analysis (limma) within the Bioconductor framework [64]. Before analysis, poor quality spots were manually flagged and filtered out. Quality of the microarray data was assessed on diagnostic plots (boxplots and density plots) and spatial images generated from the raw (non-processed) data. All twenty arrays passed these strict criteria and were included in the analyses. Intensity ratio values for all microarray spots were normalized using a within-slide global Locally Weighted Scatterplot Smoothing procedure to remove the effect of systematic variation in the microarrays; no background correction was necessary due to homogeneous hybridization. The normalized data from the arrays of each treatment group were averaged. For each comparison, differentially expressed genes were identified using FDR control with a threshold of *q *< 0.05.

Further analysis of the differentially expressed genes (for example, clustering, self-organizing maps) was performed using Bioconductor and GeneSpring 7.3 (Agilent Technologies Inc., Palo Alto, CA, USA). To gain an overall idea of the pattern of gene expression changes across the various treatments, unsupervised hierarchical cluster analysis was performed on the group of all probes differentially expressed in any of the within-treatment *Il10*^*-/- *^vs. C57 comparisons using Bioconductor.

Network, pathways and functional analyses were generated using Ingenuity Pathways Analysis (IPA, Version 5.0 or Version 6.0, Ingenuity Systems, Redwood City, CA, USA; http://www.ingenuity.com). For analysis using IPA, the full dataset (representing all IDs on the Agilent array) was uploaded for analysis, and the set of differentially expressed genes analyzed using the complete gene-list as a reference data set. For analyses where more than 800 differentially expressed genes were identified as network-eligible, a fold-change cut-off was applied to reduce this number to 800, as is recommended by IPA. The fold-change cut off for each list is reported in the results section. In the case of genes being replicated on the array, the median value of the replicates was used for IPA analysis.

Functional analysis in IPA identified biological functions and/or diseases that were most relevant to the data set. Genes from the dataset that met the FDR cutoff (*q *< 0.05) and were associated with biological functions and/or diseases in the IPA Knowledge Base were considered for the analysis. Fischer's exact test was used to calculate the probability that each biological function and/or disease assigned to that data set is due to chance alone.

Canonical pathways analysis identified those pathways from the IPA library that were most significant to the data set. Only those genes from the data set meeting the FDR cutoff (*q *< 0.05) and associated with a canonical pathway in the IPA Knowledge Base were considered for this analysis. The significance of the association between the data set and the canonical pathway was measured in two ways: 1) The ratio of the number of genes from the data set that map to the canonical pathway in question divided by the total number of genes that map to the same canonical pathway; 2) Fischer's exact test was used to calculate the probability that the association between the genes in the dataset and the canonical pathway is explained by chance alone.

Because we have used an *Il10*^*-/- *^mouse model, the "Neighborhood Explorer" feature of IPA was used to investigate genes associated with *Il10*. The Neighborhood Explorer is a network which displays all molecules that directly interact with a molecule of interest (in this case mRNA encoded by the *Il10 *gene, which is non-functional in the *Il10*^*-/- *^mice), either regulating or being regulated by that molecule, or physically interacting with it. These interactions are based on information contained within the Ingenuity Pathways Knowledge Base.

### Quantitative real-time polymerase chain reaction

Five genes showing differential expression in EF·CIF-inoculated *Il10*^*-/- *^mice (compared with similarly inoculated C57 mice) were selected for validation and their expression levels established using quantitative real-time polymerase chain reaction (qRT-PCR). cDNA was synthesized from the same total RNA samples used for the microarray analysis, using the Transcriptor First Strand cDNA Synthesis Kit (Roche Diagnostics, Mannheim, Germany). Reverse transcription was performed using 1.0 μg of total RNA and oligo-dT primers according to the manufacturer's instructions.

The PCR conditions were: 95°C for 5 min, 35 cycles at 95°C for 15 s, 63°C for 10 s and 72°C for 15 s. Melting curve analyses were performed by increasing the temperature (1°C/s) from 65°C to 95°C, with continuous fluorescence acquisition. With the exception of neutrophil cytosolic factor 4 (*Ncf4*) [59], primers for the selected genes (cytochrome P450, family 2, subfamily C, polypeptide 40 (*Cyp2c40*), carboxylesterase 2 (*Ces2*), sulfotransferase 1A (*Sult1a1*) and IFNγ (*Ifng*)) were designed using Primer 3.0 [65], with available public sequences. RefSeq IDs and primer sequences are as previously described [66]. *Ifng *and *Ncf4 *are genes associated with immune response, while *Cyp2c40, Ces2 *and *Sult1a1 *are xenobiotic metabolism genes. PCR conditions for all primers were optimized and amplicons were sequenced to confirm identity. Specificities of all PCR reactions were verified by melting curves analyses and agarose gel electrophoresis. Data were normalized against two reference genes (hypoxanthine guanine phosphoribosyl transferase 1 (*Hprt1*) and calnexin (*Canx*) [59]).

Threshold cycle (Ct) values were obtained in triplicates for each sample on the LightCycler 480 (Roche Diagnostics, Mannheim, Germany) using LightCycler 480 SYBR Green I Master (Roche Diagnostics, Mannheim, Germany) in 20 μl reactions, according to the manufacturer's protocol. Standard curves for all selected genes and reference genes were generated using serial dilutions of pooled cDNAs from all samples. LightCycler 480 Relative Quantification Software was used to calculate mRNA concentrations based on the appropriate standard curves and normalized ratios (target/reference).

### Statistical analysis

Statistical analyses of live weight, HIS, qRT-PCR and plasma SAA and cytokines were performed using GenStat (VSN International, Hemel Hempstead, UK; 9^th ^edition, 2006 or 10^th ^edition, 2007). Differences in HIS both between and within mouse strains were analyzed using an ANOVA with pooled variance. As expected, there were a high number of zero histology scores for the C57 mice and so the data for the two strains were analyzed separately. Plasma concentrations of SAA and cytokines were analyzed by ANOVA using strain and treatment as factors. Log transformed values were used for statistical analysis of the histology and SAA data. Due to the skewed nature of the data, and the large proportion of zeros observed for some of the plasma cytokine measurements, a variety of different transformations (such as log, square-root, and non-parametric rank transformations) were required across these variables. Specific transformations used are described in the results section for the cytokine analyses.

A probability value of less than 0.05 was considered significant while a probability value greater than 0.05 but lower than 0.10 was considered a trend. It must be noted that one *Il10*^*-/- *^mouse from the CIF inoculation group died during the experiment (52 days of age) for unknown reasons. This animal was not scored for histological signs of inflammation, and no data from this animal (e.g. live weight) were included in any of the analyses. Unless otherwise stated, data are presented in the text as mean ± SD.

## Authors' contributions

MPGB participated in the study design, carried out the animal trials and Pathways Analysis, and drafted the manuscript. WCM participated in the design of the study and discussion of the results. ALC supplied bacterial cultures, participated in this aspect of the study design, and generated relevant discussion. SZ performed all histological evaluations. MD performed all Bioconductor analyses and generated heat maps, BK assisted with interpretation and discussion around microarrays, KN ran RT-PCR reactions, and AJH carried out cytokine analyses. NCR supervised the study, participated in its design and coordination and helped to draft the manuscript. All authors read and approved the final manuscript.

## Supplementary Material

Additional file 1 **Pathways with genes differentially expressed in *Il10*^*-/- *^mice in response to the 1) SPF, 2) C, 3) EF, 4) CIF and 5) EF·CIF treatments when compared with similarly-inoculated C57 mice**. Fold-change and P-values for genes in the top 10 most significant canonical pathways as identified by Ingenuity Pathways Analysis (using Fischer's exact test, as shown in Table 5 within the main text). For those comparisons where there was a significant difference between *Il10*^*-/- *^and C57 mice, the text is shown in italics. Click here for file
