# Supplementary material for: Changes in colon gene expression associated with increased colon inflammation in interleukin-10 gene-deficient mice inoculated with Enterococcus species
Source: BMC Immunol. 2010 Jul 15;11:39. doi: 10.1186/1471-2172-11-39 (PMC2912833; doi:10.1186/1471-2172-11-39)
Supplement: Additional file 1 — Pathways with genes differentially expressed in Il10-/- mice in response to the 1) SPF, 2) C, 3) EF, 4) CIF and 5) EF·CIF treatments when compared with similarly-inoculated C57 mice. Fold-change and P-values for genes in the top 10 most significant canonical pathways as identified by Ingenuity Pathways Analysis (using Fischer's exact test, as shown in Table 5 within the main text). For those comparisons where there was a significant difference between Il10-/- and C57 mice, the text is shown in italics. [file 1471-2172-11-39-S1.DOC]

**Additional file 1 Table S1**

**Title: Pathways with genes differentially expressed in Il10^–/–^ mice in response to the 1) SPF, 2) C, 3) EF, 4) CIF and 5) EF·CIF treatments when compared with similarly-inoculated C57 mice.**

Description: Fold-change and P-values for genes in the top 10 most significant canonical pathways as identified by Ingenuity Pathways Analysis (using Fischer’s exact test, as shown in Table 5 within the main text). For those comparisons where there was a significant difference between Il10–/– and C57 mice, the text is shown in italics.

| Gene symbol | | Gene description | | | | | GeneBank accession number | SPF |  | C | | EF | | CIF | | EF·CIF | |
| --- | --- | --- | --- | --- | --- | --- | --- | --- | --- | --- | --- | --- | --- | --- | --- | --- | --- |
|  |  |  |  |  |  |  |  | Fold change | P-value | Fold change | P-value | Fold change | P-value | Fold change | P-value | Fold change | P-value |
| *Fatty acid metabolism* | | | | | |  | |  |  |  |  |  |  |  |  |  |  |
| ACAA1 | | acetyl-Coenzyme A acyltransferase 1 (peroxisomal 3-oxoacyl-Coenzyme A thiolase) | | | NM_130864 | | | -1.0 | 1.0 | -1.4 | 0.4 | *-2.9* | *<0.01* | *-1.8* | *<0.05* | *-2.0* | *<0.01* |
| ACAA2 | | acetyl-Coenzyme A acyltransferase 2 (mitochondrial 3-oxoacyl-Coenzyme A thiolase) | | | NM_177470 | | | -1.2 | 0.9 | -1.6 | 0.2 | *-2.8* | *<0.0001* | *-2.0* | *<0.01* | *-2.4* | *<0.0001* |
| ACAA1B | | acetyl-Coenzyme A acyltransferase 1B | | | NM_146230 | | | -1.3 | 0.9 | -2.2 | 0.2 | *-3.5* | *<0.01* | *-2.9* | *<0.01* | *-3.2* | *<0.01* |
| ACADVL | | acyl-Coenzyme A dehydrogenase, very long chain | | | NM_017366 | | | 1.1 | 0.9 | -1.2 | 0.5 | *-1.9* | *<0.0001* | *-1.6* | *<0.05* | *-1.7* | *<0.01* |
| ACOX1 | | acyl-Coenzyme A oxidase 1, palmitoyl | | | NM_015729 | | | 1.1 | 0.9 | -1.1 | 0.9 | *-2.2* | *<0.01* | -1.4 | 0.2 | *-1.7* | *<0.05* |
| ACSL3 | | acyl-CoA synthetase long-chain family member 3 | | | NM_028817 | | | -1.4 | 0.1 | *-1.7* | *<0.05* | *-2.9* | *<0.0001* | *-2.4* | *<0.0001* | *-2.5* | *<0.0001* |
| ADH5 | | alcohol dehydrogenase 5 (class III), chi polypeptide | | | NM_007410 | | | 1.1 | 0.9 | -1.4 | 0.3 | *-2.3* | *<0.0001* | *-1.8* | *<0.01* | *-1.8* | *<0.01* |
| ALDH1A1 | | aldehyde dehydrogenase 1 family, member A1 | | | NM_013467 | | | 1.1 | 1.0 | -1.3 | 0.6 | *-3.3* | *<0.0001* | *-2.7* | *<0.01* | *-2.6* | *<0.01* |
| ALDH1A7 | | aldehyde dehydrogenase family 1, subfamily A7 | | | NM_011921 | | | -1.2 | 0.8 | 1.0 | 1.0 | *-2.3* | *<0.0001* | *-1.6* | *<0.01* | *-1.8* | *<0.01* |
| CPT1A | | carnitine palmitoyltransferase 1A (liver) | | | NM_013495 | | | -1.1 | 0.9 | *-1.6* | *<0.05* | *-2.0* | *<0.0001* | *-1.7* | *<0.01* | *-1.8* | *<0.01* |
| CYP2C37 | | cytochrome P450, family 2. subfamily c, polypeptide 37 | | | NM_010001 | | | -1.0 | 1.0 | -1.3 | 0.6 | *-2.5* | *<0.0001* | -1.3 | 0.5 | -1.5 | 0.2 |
| CYP2C40 | | cytochrome P450, family 2, subfamily c, polypeptide 40 | | | NM_010004 | | | -1.5 | 0.5 | -1.5 | 0.2 | *-12.9* | *<0.0001* | *-3.9* | *<0.0001* | *-4.7* | *<0.0001* |
| CYP2C54 | | cytochrome P450, family 2, subfamily c, polypeptide 54 | | | NM_206537 | | | -1.0 | 1.0 | -1.3 | 0.3 | *-1.8* | *<0.01* | *-1.5* | *<0.05* | -1.4 | 0.1 |
| CYP2C55 | | cytochrome P450, family 2, subfamily c, polypeptide 55 | | | NM_028089 | | | 1.0 | 1.0 | -1.5 | 0.5 | *-12.5* | *<0.0001* | *-6.1* | *<0.0001* | *-8.7* | *<0.0001* |
| CYP2D10 | | cytochrome P450, family 2, subfamily d, polypeptide 10 | | | NM_010005 | | | -1.1 | 0.9 | -1.0 | 0.5 | *-1.9* | *<0.05* | -1.2 | 0.5 | -1.3 | 0.5 |
| CYP2D26 | | cytochrome P450, family 2, subfamily d, polypeptide 26 | | | NM_029562 | | | -1.4 | 0.9 | -2.2 | 0.2 | *-3.7* | *<0.01* | *-2.5* | *<0.05* | *-2.9* | *<0.05* |
| CYP2E1 | | cytochrome P450, family 2, subfamily E, polypeptide 1 | | | NM_021282 | | | -1.1 | 1.0 | -1.4 | 0.4 | *-2.7* | *<0.0001* | *-2.9* | *<0.0001* | *-2.2* | *<0.01* |
| CYP2S1 | | cytochrome P450, family 2, subfamily S, polypeptide 1 | | | NM_028775 | | | -1.1 | 0.9 | -1.3 | 0.5 | *-1.8* | *<0.01* | *-1.3* | *0.3* | *-1.8* | *<0.05* |
| CYP3A5 | | cytochrome P450, family 3, subfamily A, polypeptide 5 | | | NM_007819 | | | -1.0 | 1.0 | -1.2 | 0.6 | *-1.8* | *<0.01* | -1.0 | 1.0 | -1.3 | 0.1 |
| CYP4B1 | | cytochrome P450, family 4, subfamily B, polypeptide 1 | | | NM_007823 | | | -2.7 | 0.5 | -4.7 | 0.1 | *-15.9* | *<0.0001* | *-12.7* | *<0.0001* | *-15.1* | *<0.0001* |
| ECH1 | | enoyl Coenzyme A hydratase 1, peroxisomal | | | NM_016772 | | | -1.2 | 0.9 | -1.5 | 0.2 | *-2.0* | *<0.01* | *-1.7* | *<0.01* | *-1.8* | *<0.01* |
| EHHADH | | enoyl-Coenzyme A, hydratase/3-hydroxyacyl Coenzyme A dehydrogenase | | | NM_023737 | | | -1.3 | 0.9 | -1.8 | 0.2 | *-3.0* | *<0.01* | *-2.2* | *<0.05* | *-2.2* | *<0.05* |
| HSD17B4 | | hydroxysteroid (17-beta) dehydrogenase 4 | | | NM_008292 | | | -1.0 | 1.0 | -1.4 | 0.3 | *-2.1* | *<0.01* | *-1.9* | *<0.01* | *-1.8* | *<0.01* |
| *LPS/IL1-mediated Inhibition of RXR Function* | | |  | | | | |  |  |  |  |  |  |  |  |  |  |
| ABCB1 | | ATP-binding cassette, sub-family B (MDR/TAP), member 1 | | | NM_011076 | | | -1.3 | 0.8 | -1.5 | 0.2 | *-5.7* | *<0.0001* | -1.8 | 0.3 | *-4.5* | *<0.0001* |
| ABCB9 | | ATP-binding cassette, sub-family B (MDR/TAP), member 9 | | | NM_019875 | | | 1.0 | 1.0 | -1.2 | 0.4 | *-1.9* | *<0.0001* | *-1.7* | *<0.01* | *-2.0* | *<0.0001* |
| ACOX1 | | acyl-Coenzyme A oxidase 1, palmitoyl | | | NM_015729 | | | 1.1 | 0.9 | -1.1 | 0.9 | *-2.2* | *<0.01* | -1.4 | 0.2 | *-1.7* | *<0.05* |
| ACSL3 | | acyl-CoA synthetase long-chain family member 3 | | | NM_028817 | | | -1.4 | 0.1 | *-1.7* | *<0.05* | *-2.9* | *<0.0001* | *-2.4* | *<0.0001* | *-2.5* | *<0.0001* |
| ALDH1A1 | | aldehyde dehydrogenase 1 family, member A1 | | | NM_013467 | | | 1.1 | 1.0 | -1.3 | 0.6 | *-3.3* | *<0.0001* | *-2.7* | *<0.01* | *-2.6* | *<0.01* |
| ALDH1L1 | | aldehyde dehydrogenase 1 family, member L1 | | | NM_027406 | | | 1.1 | 0.9 | 1.7 | 0.1 | *2.9* | *<0.0001* | *2.1* | *<0.01* | *2.3* | *<0.0001* |
| ALDH6A1 | | aldehyde dehydrogenase 6 family, member A1 | | | NM_134042 | | | -1.0 | 1.0 | -1.1 | 0.7 | *-2.1* | *<0.0001* | *-1.7* | *<0.01* | *-1.9* | *<0.0001* |
| APOE | | apolipoprotein E | | | NM_009696 | | | 1.3 | 0.4 | -1.0 | 0.9 | *1.7* | *<0.0001* | *1.6* | *<0.01* | *1.6* | *<0.01* |
| CAT | | catalase | | | NM_009804 | | | -1.3 | 0.8 | -2.1 | 0.1 | *-2.1* | *<0.01* | *-2.2* | *<0.05* | -1.7 | 0.1 |
| CD14 | | CD14 molecule | | | NM_009841 | | | 1.2 | 0.8 | 1.2 | 0.5 | *2.5* | *<0.0001* | *2.2* | *<0.0001* | *2.4* | *<0.0001* |
| CPT1A | | carnitine palmitoyltransferase 1A (liver) | | | NM_013495 | | | -1.1 | 0.9 | *-1.6* | *<0.05* | *-2.0* | *<0.0001* | *-1.7* | *<0.01* | *-1.8* | *<0.01* |
| CYP3A5 | | cytochrome P450, family 3, subfamily A, polypeptide 5 | | | NM_007819 | | | -1.0 | 1.0 | -1.2 | 0.6 | *-1.8* | *<0.01* | -1.0 | 1.0 | -1.3 | 0.1 |
| FABP2 | | fatty acid binding protein 2, intestinal | | | NM_007980 | | | 1.3 | 0.8 | 1.1 | 0.2 | *-3.8* | *<0.0001* | -1.7 | 0.4 | -2.3 | 0.1 |
| FABP4 | | fatty acid binding protein 4, adipocyte | | | NM_024406 | | | -1.3 | 0.9 | -1.8 | 0.5 | *-4.3* | *<0.01* | *-4.0* | *<0.05* | *-4.2* | *<0.01* |
| FMO5 | | flavin containing monooxygenase 5 | | | NM_010232 | | | -1.1 | 0.9 | -1.5 | 0.4 | *-2.5* | *<0.01* | -1.6 | 0.1 | *-2.0* | *<0.05* |
| GSTA4 | | glutathione S-transferase A4 | | | NM_010357 | | | -1.1 | 0.9 | -1.3 | 0.2 | *-2.3* | *<0.0001* | *-1.6* | *<0.01* | *-1.7* | *<0.01* |
| GSTK1 | | glutathione S-transferase kappa 1 | | | NM_029555 | | | -1.0 | 1.0 | -1.2 | 0.4 | *-2.0* | *<0.0001* | *-1.7* | *<0.01* | *-1.9* | *<0.0001* |
| GSTM5 | | glutathione S-transferase M5 | | | NM_010358 | | | 1.0 | 0.9 | 1.1 | 0.8 | *-1.9* | *<0.0001* | -1.3 | 0.2 | -1.3 | 0.3 |
| HMGCS2 | | 3-hydroxy-3-methylglutaryl-Coenzyme A synthase 2 (mitochondrial) | | | NM_008256 | | | -1.1 | 1.0 | -1.7 | 0.3 | *-4.1* | *<0.0001* | *-3.0* | *<0.01* | *-3.3* | *<0.01* |
| HS6ST1 | | heparan sulfate 6-O-sulfotransferase 1 | | | NM_015818 | | | 1.3 | 0.5 | 1.3 | 0.3 | *1.8* | *<0.01* | *1.9* | *<0.01* | *1.6* | *<0.01* |
| IL1B | | interleukin 1, beta | | | NM_008361 | | | 1.7 | 0.6 | 1.6 | 0.4 | *9.4* | *<0.0001* | *8.0* | *<0.0001* | *8.1* | *<0.0001* |
| MAOB | | monoamine oxidase B | | | NM_172778 | | | -1.2 | 0.6 | -1.2 | 0.4 | *-3.4* | *<0.0001* | *-2.2* | *<0.0001* | *-2.5* | *<0.0001* |
| MGST3 | | microsomal glutathione S-transferase 3 | | | NM_025569 | | | -1.1 | 0.9 | -1.1 | 0.8 | *-1.7* | *<0.001* | -1.2 | 0.1 | *-1.5* | *<0.01* |
| PPARA | | peroxisome proliferator-activated receptor alpha | | | NM_011144 | | | -1.0 | 1.0 | -1.6 | 0.3 | *-3.1* | *<0.0001* | *-2.2* | *<0.01* | *-2.3* | *<0.01* |
| SULT1A1 | | sulfotransferase family, cytosolic, 1A, phenol-preferring, member 1 | | | NM_133670 | | | 1.2 | 0.9 | -1.1 | 0.9 | *-6.8* | *<0.0001* | -2.3 | 0.1 | *-4.5* | *<0.01* |
| SULT1C2 | | sulfotransferase family, cytosolic, 1C, member 2 | | | NM_026935 | | | -1.1 | 1.0 | -1.4 | 0.4 | *-5.7* | *<0.0001* | *-3.4* | *<0.0001* | *-5.0* | *<0.0001* |
| SULT1D1 | | sulfotransferase family 1D, member 1 | | | NM_016771 | | | -1.1 | 1.0 | -1.4 | 0.2 | *-3.6* | *<0.0001* | *-2.3* | *<0.0001* | *-2.9* | *<0.0001* |
| TNF | | tumor necrosis factor (TNF superfamily, member 2) | | | NM_013693 | | | 1.1 | 0.9 | 1.1 | 0.5 | *2.4* | *<0.0001* | *2.0* | *<0.0001* | *2.3* | *<0.0001* |
| TNFRSF1A | | tumor necrosis factor receptor superfamily, member 1A | | | NM_011609 | | | 1.2 | 0.9 | 1.2 | 0.5 | *1.7* | *<0.01* | *1.5* | *<0.05* | *1.7* | *<0.01* |
| TNFRSF1B | | tumor necrosis factor receptor superfamily, member 1B | | | NM_011610 | | | 1.2 | 0.7 | 1.1 | 0.6 | *2.1* | *<0.0001* | 1.6 | 0.3 | 1.5 | 0.4 |
| *Tryptophan Metabolism* | | | | | | |  |  |  |  |  |  |  |  |  |  |  |
| ABP1 | | amiloride binding protein 1 (amine oxidase (copper-containing)) | | | NM_029638 | | | -1.1 | 1.0 | -1.7 | 0.3 | *-4.0* | *<0.0001* | *-3.2* | *<0.01* | *-3.1* | *<0.01* |
| ACAA1 | | acetyl-Coenzyme A acyltransferase 1 (peroxisomal 3-oxoacyl-Coenzyme A thiolase) | | | NM_130864 | | | -1.0 | 1.0 | -1.4 | 0.4 | *-2.9* | *<0.01* | *-1.8* | *<0.05* | *-2.0* | *<0.01* |
| ALDH1A1 | | aldehyde dehydrogenase 1 family, member A1 | | | NM_013467 | | | 1.1 | 1.0 | -1.3 | 0.6 | *-3.3* | *<0.0001* | *-2.7* | *<0.01* | *-2.6* | *<0.01* |
| ALDH1A7 | | aldehyde dehydrogenase family 1, subfamily A7 | | | NM_011921 | | | -1.2 | 0.8 | 1.0 | 1.0 | *-2.3* | *<0.0001* | *-1.6* | *<0.01* | *-1.8* | *<0.01* |
| AOC3 | | amine oxidase, copper containing 3 (vascular adhesion protein 1) | | | NM_009675 | | | -1.1 | 1.0 | -1.4 | 0.2 | *-2.3* | *<0.0001* | *-2.4* | *<0.0001* | *-2.5* | *<0.0001* |
| CAT | | catalase | | | NM_009804 | | | -1.3 | 0.8 | -2.1 | 0.1 | *-2.1* | *<0.05* | *-2.2* | *<0.05* | *-1.7* | *0.1* |
| CYP2C37 | | cytochrome P450, family 2. subfamily c, polypeptide 37 | | | NM_010001 | | | -1.0 | 1.0 | -1.3 | 0.6 | *-2.5* | *<0.0001* | -1.3 | 0.5 | -1.5 | 0.2 |
| CYP2C40 | | cytochrome P450, family 2, subfamily c, polypeptide 40 | | | NM_010004 | | | -1.5 | 0.5 | -1.5 | 0.2 | *-12.9* | *<0.0001* | *-3.9* | *<0.0001* | *-4.7* | *<0.0001* |
| CYP2C54 | | cytochrome P450, family 2, subfamily c, polypeptide 54 | | | NM_206537 | | | -1.0 | 1.0 | -1.3 | 0.3 | *-1.8* | *<0.01* | *-1.5* | *<0.05* | *-1.4* | *0.1* |
| CYP2C55 | | cytochrome P450, family 2, subfamily c, polypeptide 55 | | | NM_028089 | | | 1.0 | 1.0 | -1.5 | 0.5 | *-12.5* | *<0.0001* | *-6.1* | *<0.0001* | *-8.7* | *<0.0001* |
| CYP2D10 | | cytochrome P450, family 2, subfamily d, polypeptide 10 | | | NM_010005 | | | -1.1 | 0.9 | -1.0 | 0.5 | *-1.9* | *<0.05* | -1.2 | 0.5 | -1.3 | 0.5 |
| CYP2D26 | | cytochrome P450, family 2, subfamily d, polypeptide 26 | | | NM_029562 | | | -1.4 | 0.9 | -2.2 | 0.2 | *-3.7* | *<0.01* | *-2.5* | *<0.05* | *-2.9* | *<0.05* |
| CYP2E1 | | cytochrome P450, family 2, subfamily E, polypeptide 1 | | | NM_021282 | | | -1.1 | 1.0 | -1.4 | 0.4 | *-2.7* | *<0.0001* | *-2.9* | *<0.0001* | *-2.2* | *<0.01* |
| CYP2S1 | | cytochrome P450, family 2, subfamily S, polypeptide 1 | | | NM_028775 | | | -1.1 | 0.9 | -1.3 | 0.5 | *-1.8* | *<0.01* | -1.3 | 0.3 | *-1.8* | *<0.05* |
| CYP3A5 | | cytochrome P450, family 3, subfamily A, polypeptide 5 | | | NM_007819 | | | -1.0 | 1.0 | -1.2 | 0.6 | *-1.8* | *<0.01* | -1.0 | 1.0 | -1.3 | 0.1 |
| CYP4B1 | | cytochrome P450, family 4, subfamily B, polypeptide 1 | | | NM_007823 | | | -2.7 | 0.5 | -4.7 | 0.1 | *-15.9* | *<0.0001* | *-12.7* | *<0.0001* | *-15.1* | *<0.0001* |
| DDC | | dopa decarboxylase (aromatic L-amino acid decarboxylase) | | | NM_016672 | | | -1.2 | 0.6 | -1.2 | 0.5 | *-1.9* | *<0.0001* | *-1.4* | *0.2* | *-1.6* | *0.1* |
| ECH1 | | enoyl Coenzyme A hydratase 1, peroxisomal | | | NM_016772 | | | -1.2 | 0.9 | -1.5 | 0.2 | *-2.0* | *<0.01* | *-1.7* | *<0.01* | *-1.8* | *<0.01* |
| EHHADH | | enoyl-Coenzyme A, hydratase/3-hydroxyacyl Coenzyme A dehydrogenase | | | NM_023737 | | | -1.3 | 0.9 | -1.8 | 0.2 | *-3.0* | *<0.01* | *-2.2* | *<0.05* | *-2.2* | *<0.05* |
| HSD17B4 | | hydroxysteroid (17-beta) dehydrogenase 4 | | | NM_008292 | | | -1.0 | 1.0 | -1.4 | 0.3 | *-2.1* | *<0.01* | *-1.9* | *<0.01* | *-1.8* | *<0.01* |
| INDO | | indoleamine-pyrrole 2,3 dioxygenase | | | NM_008324 | | | *4.0* | *<0.01* | *4.8* | *<0.01* | *12.6* | *<0.0001* | *11.7* | *<0.0001* | *11.3* | *<0.0001* |
| MAOB | | monoamine oxidase B | | | NM_172778 | | | -1.2 | 0.6 | -1.2 | 0.4 | *-3.4* | *<0.0001* | *-2.2* | *<0.0001* | *-2.5* | *<0.0001* |
| WARS | | tryptophanyl-tRNA synthetase | | | NM_011710 | | | 1.2 | 0.7 | 1.4 | 0.4 | *3.2* | *<0.0001* | *2.0* | *<0.01* | *2.3* | *<0.01* |
| *β-Alanine Metabolism* | | | |  | | | |  |  |  |  |  |  |  |  |  |  |
| ABAT | | 4-aminobutyrate aminotransferase | | | NM_172961 | | | -1.1 | 0.9 | 1.1 | 0.9 | *-1.8* | *<0.01* | *-1.6* | *<0.05* | *-2.0* | *<0.01* |
| ABP1 | | amiloride binding protein 1 (amine oxidase (copper-containing)) | | | NM_029638 | | | -1.1 | 1.0 | -1.7 | 0.3 | *-4.0* | *<0.0001* | *-3.2* | *<0.01* | *-3.1* | *<0.01* |
| ACADVL | | acyl-Coenzyme A dehydrogenase, very long chain | | | NM_017366 | | | 1.1 | 0.9 | -1.2 | 0.5 | *-1.9* | *<0.0001* | *-1.6* | *<0.05* | *-1.7* | *<0.01* |
| ALDH2 | | aldehyde dehydrogenase 2 family (mitochondrial) | | | NM_009656 | | | 1.0 | 0.9 | 1.1 | 0.6 | *-1.6* | *<0.0001* | *-1.5* | *<0.01* | *-1.6* | *<0.01* |
| ALDH1A1 | | aldehyde dehydrogenase 1 family, member A1 | | | NM_013467 | | | 1.1 | 1.0 | -1.3 | 0.6 | *-3.3* | *<0.0001* | *-2.7* | *<0.01* | *-2.6* | *<0.01* |
| ALDH1A7 | | aldehyde dehydrogenase family 1, subfamily A7 | | | NM_011921 | | | -1.2 | 0.8 | 1.0 | 1.0 | *-2.3* | *<0.0001* | *-1.6* | *<0.01* | *-1.8* | *<0.01* |
| ALDH6A1 | | aldehyde dehydrogenase 6 family, member A1 | | | NM_134042 | | | -1.0 | 1.0 | -1.1 | 0.7 | *-2.1* | *<0.0001* | *-1.7* | *<0.01* | *-1.9* | *<0.0001* |
| AOC3 | | amine oxidase, copper containing 3 (vascular adhesion protein 1) | | | NM_009675 | | | -1.1 | 1.0 | -1.4 | 0.2 | *-2.3* | *<0.0001* | *-2.4* | *<0.0001* | *-2.5* | *<0.0001* |
| DPYD | | dihydropyrimidine dehydrogenase | | | NM_170778 | | | 1.0 | 1.0 | -1.2 | 0.6 | *-1.8* | *<0.01* | *-1.5* | *<0.05* | *-1.6* | *<0.01* |
| DPYSL3 | | dihydropyrimidinase-like 3 | | | NM_009468 | | | -1.3 | 0.5 | -1.2 | 0.5 | *-1.5* | *<0.05* | *-1.5* | *<0.05* | *-1.4* | *<0.05* |
| ECH1 | | enoyl Coenzyme A hydratase 1, peroxisomal | | | NM_016772 | | | -1.2 | 0.9 | -1.5 | 0.2 | *-2.0* | *<0.01* | *-1.7* | *<0.01* | *-1.8* | *<0.01* |
| EHHADH | | enoyl-Coenzyme A, hydratase/3-hydroxyacyl Coenzyme A dehydrogenase | | | NM_023737 | | | -1.3 | 0.9 | -1.8 | 0.2 | *-3.0* | *<0.01* | *-2.2* | *<0.05* | *-2.2* | *<0.05* |
| HADHB | | hydroxyacyl-Coenzyme A dehydrogenase/3-ketoacyl-Coenzyme A thiolase/enoyl-Coenzyme A hydratase (trifunctional protein), beta subunit | | | NM_145558 | | | -1.0 | 1.0 | -1.3 | 0.3 | *-1.7* | *<0.01* | *-1.4* | *<0.05* | *-1.5* | *<0.01* |
| MLYCD | | malonyl-CoA decarboxylase | | | NM_019966 | | | 1.0 | 1.0 | -1.1 | 0.8 | *-1.6* | *<0.01* | *-1.5* | *<0.01* | *-1.4* | *<0.01* |
| UPB1 | | ureidopropionase, beta | | | NM_133995 | | | 1.1 | 0.9 | 1.0 | 1.0 | *-1.5* | *<0.05* | *-1.5* | *<0.05* | *-1.6* | *<0.01* |
| *Valine, Leucine and Isoleucine Degradation* | | | |  | | | |  |  |  |  |  |  |  |  |  |  |
| ABAT | | 4-aminobutyrate aminotransferase | | | NM_172961 | | | -1.1 | 0.9 | 1.1 | 0.9 | *-1.8* | *<0.01* | *-1.6* | *<0.05* | *-2.0* | *<0.01* |
| ACAA1 | | acetyl-Coenzyme A acyltransferase 1 (peroxisomal 3-oxoacyl-Coenzyme A thiolase) | | | NM_130864 | | | -1.0 | 1.0 | -1.4 | 0.4 | *-2.9* | *<0.01* | *-1.8* | *<0.05* | *-2.0* | *<0.01* |
| ACAA2 | | acetyl-Coenzyme A acyltransferase 2 (mitochondrial 3-oxoacyl-Coenzyme A thiolase) | | | NM_177470 | | | -1.2 | 0.9 | -1.6 | 0.2 | *-2.8* | *<0.0001* | *-2.0* | *<0.01* | *-2.4* | *<0.0001* |
| ACAA1B | | acetyl-Coenzyme A acyltransferase 1B | | | NM_146230 | | | -1.3 | 0.9 | -2.2 | 0.2 | *-3.5* | *<0.01* | *-2.9* | *<0.01* | *-3.2* | *<0.01* |
| ACADM | | acyl-Coenzyme A dehydrogenase, C-4 to C-12 straight chain | | | NM_007382 | | | 1.0 | 1.0 | -1.1 | 0.8 | *-1.6* | *<0.01* | *-1.4* | *<0.05* | *-1.7* | *<0.01* |
| ACADVL | | acyl-Coenzyme A dehydrogenase, very long chain | | | NM_017366 | | | 1.1 | 0.9 | -1.2 | 0.5 | *-1.9* | *<0.0001* | *-1.6* | *<0.05* | *-1.7* | *<0.01* |
| ALDH2 | | aldehyde dehydrogenase 2 family (mitochondrial) | | | NM_009656 | | | 1.0 | 0.9 | 1.1 | 0.6 | *-1.6* | *<0.0001* | *-1.5* | *<0.01* | *-1.6* | *<0.01* |
| ALDH1A1 | | aldehyde dehydrogenase 1 family, member A1 | | | NM_013467 | | | 1.1 | 1.0 | -1.3 | 0.6 | *-3.3* | *<0.0001* | *-2.7* | *<0.01* | *-2.6* | *<0.01* |
| ALDH1A7 | | aldehyde dehydrogenase family 1, subfamily A7 | | | NM_011921 | | | -1.2 | 0.8 | 1.0 | 1.0 | *-2.3* | *<0.0001* | *-1.6* | *<0.01* | *-1.8* | *<0.01* |
| ALDH6A1 | | aldehyde dehydrogenase 6 family, member A1 | | | NM_134042 | | | -1.0 | 1.0 | -1.1 | 0.7 | *-2.1* | *<0.0001* | *-1.7* | *<0.01* | *-1.9* | *<0.0001* |
| ECH1 | | enoyl Coenzyme A hydratase 1, peroxisomal | | | NM_016772 | | | -1.2 | 0.9 | -1.5 | 0.2 | *-2.0* | *<0.01* | *-1.7* | *<0.01* | *-1.8* | *<0.01* |
| EHHADH | | enoyl-Coenzyme A, hydratase/3-hydroxyacyl Coenzyme A dehydrogenase | | | NM_023737 | | | -1.3 | 0.9 | -1.8 | 0.2 | *-3.0* | *<0.01* | *-2.2* | *<0.05* | *-2.2* | *<0.05* |
| HADH | | hydroxyacyl-Coenzyme A dehydrogenase | | | NM_008212 | | | 1.1 | 0.9 | -1.0 | 1.0 | *-1.7* | *<0.01* | -1.3 | 0.2 | *-1.6* | *<0.01* |
| HADHB | | hydroxyacyl-Coenzyme A dehydrogenase/3-ketoacyl-Coenzyme A thiolase/enoyl-Coenzyme A hydratase (trifunctional protein), beta subunit | | | NM_145558 | | | -1.0 | 1.0 | -1.3 | 0.3 | *-1.7* | *<0.01* | *-1.4* | *<0.05* | *-1.5* | *<0.01* |
| HMGCS2 | | 3-hydroxy-3-methylglutaryl-Coenzyme A synthase 2 (mitochondrial) | | | NM_008256 | | | -1.1 | 1.0 | -1.7 | 0.3 | *-4.1* | *<0.0001* | *-3.0* | *<0.01* | *-3.3* | *<0.01* |
| HSD17B4 | | hydroxysteroid (17-beta) dehydrogenase 4 | | | NM_008292 | | | -1.0 | 1.0 | -1.4 | 0.3 | *-2.1* | *<0.01* | *-1.9* | *<0.01* | *-1.8* | *<0.01* |
| *Propanoate metabolism* | | | | |  | | |  |  |  |  |  |  |  |  |  |  |
| ABAT | | 4-aminobutyrate aminotransferase | | | NM_172961 | | | -1.1 | 0.9 | 1.1 | 0.9 | *-1.8* | *<0.01* | *-1.6* | *<0.05* | *-2.0* | *<0.01* |
| ACAA1 | | acetyl-Coenzyme A acyltransferase 1 (peroxisomal 3-oxoacyl-Coenzyme A thiolase) | | | NM_130864 | | | -1.0 | 1.0 | -1.4 | 0.4 | *-2.9* | *<0.01* | *-1.8* | *<0.05* | *-2.0* | *<0.01* |
| ACACB | | acetyl-Coenzyme A carboxylase beta | | | BC022940 | | | -1.0 | 1.0 | -1.6 | 0.1 | *-2.2* | *<0.01* | *-2.1* | *<0.01* | *-2.0* | *<0.01* |
| ACADVL | | acyl-Coenzyme A dehydrogenase, very long chain | | | NM_017366 | | | 1.1 | 0.9 | -1.2 | 0.5 | *-1.9* | *<0.0001* | *-1.6* | *<0.05* | *-1.7* | *<0.01* |
| ACSL3 | | acyl-CoA synthetase long-chain family member 3 | | | NM_028817 | | | -1.4 | 0.1 | *-1.7* | *<0.05* | *-2.9* | *<0.0001* | *-2.4* | *<0.0001* | *-2.5* | *<0.0001* |
| ACSS1 | | acyl-CoA synthetase short-chain family member 1 | | | NM_080575 | | | -1.0 | 1.0 | -1.1 | 0.8 | *-2.6* | *<0.0001* | *-2.0* | *<0.01* | *-2.2* | *<0.01* |
| ALDH2 | | aldehyde dehydrogenase 2 family (mitochondrial) | | | NM_009656 | | | 1.0 | 0.9 | 1.1 | 0.6 | *-1.6* | *<0.0001* | *-1.5* | *<0.01* | *-1.6* | *<0.01* |
| ALDH1A1 | | aldehyde dehydrogenase 1 family, member A1 | | | NM_013467 | | | 1.1 | 1.0 | -1.3 | 0.6 | *-3.3* | *<0.0001* | *-2.7* | *<0.01* | *-2.6* | *<0.01* |
| ALDH1A7 | | aldehyde dehydrogenase family 1, subfamily A7 | | | NM_011921 | | | -1.2 | 0.8 | 1.0 | 1.0 | *-2.3* | *<0.0001* | *-1.6* | *<0.01* | *-1.8* | *<0.01* |
| ALDH6A1 | | aldehyde dehydrogenase 6 family, member A1 | | | NM_134042 | | | -1.0 | 1.0 | -1.1 | 0.7 | *-2.1* | *<0.0001* | *-1.7* | *<0.01* | *-1.9* | *<0.0001* |
| ECH1 | | enoyl Coenzyme A hydratase 1, peroxisomal | | | NM_016772 | | | -1.2 | 0.9 | -1.5 | 0.2 | *-2.0* | *<0.01* | *-1.7* | *<0.01* | *-1.8* | *<0.01* |
| EHHADH | | enoyl-Coenzyme A, hydratase/3-hydroxyacyl Coenzyme A dehydrogenase | | | NM_023737 | | | -1.3 | 0.9 | -1.8 | 0.2 | *-3.0* | *<0.01* | *-2.2* | *<0.05* | *-2.2* | *<0.05* |
| HADHB | | hydroxyacyl-Coenzyme A dehydrogenase/3-ketoacyl-Coenzyme A thiolase/enoyl-Coenzyme A hydratase (trifunctional protein), beta subunit | | | NM_145558 | | | -1.0 | 1.0 | -1.3 | 0.3 | *-1.7* | *<0.01* | *-1.4* | *<0.05* | *-1.5* | *<0.01* |
| LDHB | | lactate dehydrogenase B | | | NM_008492 | | | -1.3 | 0.3 | -1.3 | 0.2 | *-2.4* | *<0.0001* | *-1.9* | *<0.0001* | *-2.1* | *<0.0001* |
| MLYCD | | malonyl-CoA decarboxylase | | | NM_019966 | | | 1.0 | 1.0 | -1.1 | 0.8 | *-1.6* | *<0.01* | *-1.5* | *<0.01* | *-1.4* | *<0.01* |
| *Metabolism of Xenobiotics by Cytochrome P450* | | | | |  | | |  |  |  |  |  |  |  |  |  |  |
| ADH5 | | alcohol dehydrogenase 5 (class III), chi polypeptide | | | NM_007410 | | | 1.1 | 0.9 | -1.4 | 0.3 | *-2.3* | *<0.0001* | *-1.8* | *<0.01* | *-1.8* | *<0.01* |
| ALDH1L1 | | aldehyde dehydrogenase 1 family, member L1 | | | NM_027406 | | | 1.1 | 0.9 | 1.7 | 0.1 | *2.9* | *<0.0001* | *2.1* | *<0.01* | *2.3* | *<0.0001* |
| CYP2C37 | | cytochrome P450, family 2. subfamily c, polypeptide 37 | | | NM_010001 | | | -1.0 | 1.0 | -1.3 | 0.6 | *-2.5* | *<0.0001* | -1.3 | 0.5 | -1.5 | 0.2 |
| CYP2C40 | | cytochrome P450, family 2, subfamily c, polypeptide 40 | | | NM_010004 | | | -1.5 | 0.5 | -1.5 | 0.2 | *-12.9* | *<0.0001* | *-3.9* | *<0.0001* | *-4.7* | *<0.0001* |
| CYP2C54 | | cytochrome P450, family 2, subfamily c, polypeptide 54 | | | NM_206537 | | | -1.0 | 1.0 | -1.3 | 0.3 | *-1.8* | *<0.01* | *-1.5* | *<0.05* | -1.4 | 0.1 |
| CYP2C55 | | cytochrome P450, family 2, subfamily c, polypeptide 55 | | | NM_028089 | | | 1.0 | 1.0 | -1.5 | 0.5 | *-12.5* | *<0.0001* | *-6.1* | *<0.0001* | *-8.7* | *<0.0001* |
| CYP2D10 | | cytochrome P450, family 2, subfamily d, polypeptide 10 | | | NM_010005 | | | -1.1 | 0.9 | -1.0 | 0.5 | *-1.9* | *<0.05* | -1.2 | 0.5 | -1.3 | 0.5 |
| CYP2D26 | | cytochrome P450, family 2, subfamily d, polypeptide 26 | | | NM_029562 | | | -1.4 | 0.9 | -2.2 | 0.2 | *-3.7* | *<0.01* | *-2.5* | *<0.05* | *-2.9* | *<0.05* |
| CYP2E1 | | cytochrome P450, family 2, subfamily E, polypeptide 1 | | | NM_021282 | | | -1.1 | 1.0 | -1.4 | 0.4 | *-2.7* | *<0.0001* | *-2.9* | *<0.0001* | *-2.2* | *<0.01* |
| CYP2S1 | | cytochrome P450, family 2, subfamily S, polypeptide 1 | | | NM_028775 | | | -1.1 | 0.9 | -1.3 | 0.5 | *-1.8* | *<0.01* | -1.3 | 0.3 | *-1.8* | *<0.05* |
| CYP3A5 | | cytochrome P450, family 3, subfamily A, polypeptide 5 | | | NM_007819 | | | -1.0 | 1.0 | -1.2 | 0.6 | *-1.8* | *<0.01* | -1.0 | 1.0 | -1.3 | 0.1 |
| CYP4B1 | | cytochrome P450, family 4, subfamily B, polypeptide 1 | | | NM_007823 | | | -2.7 | 0.5 | -4.7 | 0.1 | *-15.9* | *<0.0001* | *-12.7* | *<0.0001* | *-15.1* | *<0.0001* |
| GSTA4 | | glutathione S-transferase A4 | | | NM_010357 | | | -1.1 | 0.9 | -1.3 | 0.2 | *-2.3* | *<0.0001* | *-1.6* | *<0.01* | *-1.7* | *<0.01* |
| GSTK1 | | glutathione S-transferase kappa 1 | | | NM_029555 | | | -1.0 | 1.0 | -1.2 | 0.4 | *-2.0* | *<0.0001* | *-1.7* | *<0.01* | *-1.9* | *<0.0001* |
| GSTM5 | | glutathione S-transferase M5 | | | NM_010358 | | | 1.0 | 0.9 | 1.1 | 0.8 | *-1.9* | *<0.0001* | -1.3 | 0.2 | -1.3 | 0.3 |
| GSTM3 (includes EG:14864) | | glutathione S-transferase, mu 3 | | | NM_010359 | | | -1.0 | 1.0 | 1.0 | 0.9 | *-1.7* | *<0.01* | *-1.5* | *<0.05* | *-1.4* | *<0.05* |
| UGT1A1 | | UDP glucuronosyltransferase 1 family, polypeptide A1 | | | NM_201645 | | | 1.0 | 1.0 | -1.2 | 0.6 | *-2.2* | *<0.01* | *-1.7* | *<0.05* | *-1.9* | *<0.01* |
| UGT2B5 | | UDP glucuronosyltransferase 2 family, polypeptide B5 | | | NM_009467 | | | 1.3 | 0.9 | -1.6 | 0.3 | *-2.3* | *<0.01* | *-2.0* | *<0.05* | *-2.0* | *<0.05* |
| UGT2B37 | | UDP glucuronosyltransferase 2 family, polypeptide B37 | | | NM_053215 | | | 1.2 | 0.9 | -1.4 | 0.4 | *-2.3* | *<0.01* | *-1.8* | *<0.05* | *-1.9* | *<0.05* |
| *Antigen Presentation Pathway* | | | | |  | | |  |  |  |  |  |  |  |  |  |  |
| CD74 | | CD74 molecule, major histocompatibility complex, class II invariant chain | | | NM_010545 | | | *3.0* | *0.0* | *3.1* | *<0.01* | *6.3* | *<0.0001* | *6.0* | *<0.0001* | *5.7* | *<0.0001* |
| HLA-DMA | | major histocompatibility complex, class II, DM alpha | | | NM_010386 | | | *1.7* | *0.0* | 1.4 | 0.2 | *3.9* | *<0.0001* | *2.7* | *<0.0001* | *2.9* | *<0.0001* |
| H2-DMB1 | | histocompatibility 2, class II, locus Mb1 | | | NM_010387 | | | *3.7* | *0.0* | *3.4* | *<0.01* | *9.1* | *<0.0001* | *8.3* | *<0.0001* | *7.1* | *<0.0001* |
| HLA-DQA1 | | major histocompatibility complex, class II, DQ alpha 1 | | | NM_010378 | | | *1.9* | *0.0* | *2.1* | *<0.01* | *4.8* | *<0.0001* | *4.7* | *<0.0001* | *4.3* | *<0.0001* |
| H2-AB1 | | histocompatibility 2, class II antigen A, beta 1 | | | NM_207105 | | | *2.5* | *0.0* | *2.6* | *<0.001* | *6.7* | *<0.0001* | *6.2* | *<0.0001* | *5.2* | *<0.0001* |
| HLA-DRB1 | | major histocompatibility complex, class II, DR beta 1 | | | NM_010382 | | | *2.6* | *0.0* | *2.2* | *<0.01* | *5.9* | *<0.0001* | *5.5* | *<0.0001* | *4.6* | *<0.0001* |
| PSMB8 | | proteasome (prosome, macropain) subunit, beta type, 8 (large multifunctional peptidase 7) | | | NM_010724 | | | 1.7 | 0.2 | 1.3 | 0.4 | *3.3* | *<0.0001* | *3.5* | *<0.0001* | *3.9* | *<0.0001* |
| PSMB9 | | proteasome (prosome, macropain) subunit, beta type, 9 (large multifunctional peptidase 2) | | | NM_013585 | | | 1.5 | 0.5 | 1.4 | 0.4 | *3.5* | *<0.0001* | *3.5* | *<0.0001* | *3.8* | *<0.0001* |
| TAP1 | | transporter 1, ATP-binding cassette, sub-family B (MDR/TAP) | | | NM_013683 | | | 1.1 | 0.9 | 1.2 | 0.5 | *3.1* | *<0.0001* | *2.6* | *<0.0001* | *2.5* | *<0.0001* |
| TAP2 | | transporter 2, ATP-binding cassette, sub-family B (MDR/TAP) | | | NM_011530 | | | 1.1 | 0.8 | 1.2 | 0.5 | *1.7* | *<0.0001* | *1.6* | *<0.01* | *1.7* | *<0.0001* |
| *Fatty Acid Elongation in Mitochondria* | | | | |  | | |  |  |  |  |  |  |  |  |  |  |
| ACAA1 | | acetyl-Coenzyme A acyltransferase 1 (peroxisomal 3-oxoacyl-Coenzyme A thiolase) | | | NM_130864 | | | -1.0 | 1.0 | -1.4 | 0.4 | *-2.9* | *<0.01* | *-1.8* | *<0.05* | *-2.0* | *<0.01* |
| ACAA2 | | acetyl-Coenzyme A acyltransferase 2 (mitochondrial 3-oxoacyl-Coenzyme A thiolase) | | | NM_177470 | | | -1.2 | 0.9 | -1.6 | 0.2 | *-2.8* | *<0.0001* | *-2.0* | *<0.01* | *-2.4* | *<0.0001* |
| ACAA1B | | acetyl-Coenzyme A acyltransferase 1B | | | NM_146230 | | | -1.3 | 0.9 | -2.2 | 0.2 | *-3.5* | *<0.01* | *-2.9* | *<0.01* | *-3.2* | *<0.01* |
| ECH1 | | enoyl Coenzyme A hydratase 1, peroxisomal | | | NM_016772 | | | -1.2 | 0.9 | -1.5 | 0.2 | *-2.0* | *<0.01* | *-1.7* | *<0.01* | *-1.8* | *<0.01* |
| EHHADH | | enoyl-Coenzyme A, hydratase/3-hydroxyacyl Coenzyme A dehydrogenase | | | NM_023737 | | | -1.3 | 0.9 | -1.8 | 0.2 | *-3.0* | *<0.01* | *-2.2* | *<0.05* | *-2.2* | *<0.05* |
| HADHB | | hydroxyacyl-Coenzyme A dehydrogenase/3-ketoacyl-Coenzyme A thiolase/enoyl-Coenzyme A hydratase (trifunctional protein), beta subunit | | | NM_145558 | | | -1.0 | 1.0 | -1.3 | 0.3 | *-1.7* | *<0.01* | *-1.4* | *<0.05* | *-1.5* | *<0.01* |
| HSD17B4 | | hydroxysteroid (17-beta) dehydrogenase 4 | | | NM_008292 | | | -1.0 | 1.0 | -1.4 | 0.3 | *-2.1* | *<0.01* | *-1.9* | *<0.01* | *-1.8* | *<0.01* |
| *Glycolysis/Gluconeogenesis* | | | | |  | | |  |  |  |  |  |  |  |  |  |  |
| ACSL3 | acyl-CoA synthetase long-chain family member 3 | | | | NM_028817 | | | -1.4 | 0.1 | *-1.7* | *<0.05* | *-2.9* | *<0.0001* | *-2.4* | *<0.0001* | *-2.5* | *<0.0001* |
| ACSS1 | acyl-CoA synthetase short-chain family member 1 | | | | NM_080575 | | | -1.0 | 1.0 | -1.1 | 0.8 | *-2.6* | *<0.0001* | *-2.0* | *<0.01* | *-2.2* | *<0.01* |
| ADH5 | alcohol dehydrogenase 5 (class III), chi polypeptide | | | | NM_007410 | | | 1.1 | 0.9 | -1.4 | 0.3 | *-2.3* | *<0.0001* | *-1.8* | *<0.01* | *-1.8* | *<0.01* |
| ADH1C (includes EG:126) | alcohol dehydrogenase 1C (class I), gamma polypeptide | | | | NM_007409 | | | -1.4 | 0.7 | 1.4 | 0.5 | -1.6 | 0.1 | -1.2 | 0.7 | *-1.8* | *<0.05* |
| ALDH2 | aldehyde dehydrogenase 2 family (mitochondrial) | | | | NM_009656 | | | 1.0 | 0.9 | 1.1 | 0.6 | *-1.6* | *<0.0001* | *-1.5* | *<0.01* | *-1.6* | *<0.01* |
| ALDH1A1 | aldehyde dehydrogenase 1 family, member A1 | | | | NM_013467 | | | 1.1 | 1.0 | -1.3 | 0.6 | *-3.3* | *<0.0001* | *-2.7* | *<0.01* | *-2.6* | *<0.01* |
| ALDH1A7 | aldehyde dehydrogenase family 1, subfamily A7 | | | | NM_011921 | | | -1.2 | 0.8 | 1.0 | 1.0 | *-2.3* | *<0.0001* | *-1.6* | *<0.01* | *-1.8* | *<0.01* |
| ALDH1L1 | aldehyde dehydrogenase 1 family, member L1 | | | | NM_027406 | | | 1.1 | 0.9 | 1.7 | 0.1 | *2.9* | *<0.0001* | *2.1* | *<0.01* | *2.3* | *<0.0001* |
| ALDOB | aldolase B, fructose-bisphosphate | | | | NM_144903 | | | -1.1 | 1.0 | -1.1 | 0.9 | *-2.6* | *<0.01* | -1.7 | 0.2 | *-2.7* | *<0.01* |
| ENO1 | enolase 1, (alpha) | | | | NM_023119 | | | -1.0 | 1.0 | 1.1 | 0.6 | *1.6* | *<0.01* | *1.5* | *0.0* | *1.6* | *<0.01* |
| ENO3 | enolase 3 (beta, muscle) | | | | NM_007933 | | | -1.5 | 0.4 | -1.0 | 1.0 | -1.4 | 0.1 | -1.1 | 0.8 | *-1.5* | *0.1* |
| HK2 | hexokinase 2 | | | | NM_013820 | | | 1.1 | 0.9 | *2.0* | *<0.05* | *2.4* | *<0.0001* | *2.9* | *<0.0001* | *3.2* | *<0.0001* |
| HKDC1 | hexokinase domain containing 1 | | | | NM_145419 | | | 1.2 | 0.9 | -2.1 | 0.1 | *-3.4* | *<0.0001* | *-2.9* | *<0.01* | *-3.6* | *<0.0001* |
| LDHB | lactate dehydrogenase B | | | | NM_008492 | | | -1.3 | 0.3 | -1.3 | 0.2 | *-2.4* | *<0.0001* | *-1.9* | *<0.0001* | *-2.1* | *<0.0001* |
| PGK2 | phosphoglycerate kinase 2 | | | | NM_031190 | | | -1.1 | 0.9 | 1.1 | 0.9 | -1.6 | 0.1 | -1.5 | 0.1 | *-1.7* | *<0.05* |
| PGM1 | phosphoglucomutase 1 | | | | NM_028132 | | | -1.1 | 0.9 | -1.4 | 0.3 | *-1.6* | *<0.01* | -1.2 | 0.3 | *-1.5* | *<0.05* |
